# Biochemical and pathological changes result from mutated Caveolin-3 in muscle

**DOI:** 10.1186/s13395-018-0173-y

**Published:** 2018-08-28

**Authors:** José Andrés González Coraspe, Joachim Weis, Mary E. Anderson, Ute Münchberg, Kristina Lorenz, Stephan Buchkremer, Stephanie Carr, René Peiman Zahedi, Eva Brauers, Hannah Michels, Yoshihide Sunada, Hanns Lochmüller, Kevin P. Campbell, Erik Freier, Denisa Hathazi, Andreas Roos

**Affiliations:** 1 Institute of Neuropathology, RWTH Aachen University Hospital, Pauwelsstr. 30, 52074 Aachen, Germany; 2 Howard Hughes Medical Institute, Departments of Molecular Physiology and Biophysics, of Neurology, University of Iowa, Iowa City, IA 52242 USA; 3 Biomedical Research Department, Tissue Omics group, Leibniz-Institut für Analytische Wissenschaften - ISAS - e.V, Otto-Hahn-Str. 6b, 44227 Dortmund, Germany; 4 Institute of Genetic Medicine, International Centre for Life, Central Parkway, Newcastle upon Tyne, England, UK; 5 Gerald Bronfman Department of Oncology, Jewish General Hospital, McGill University, Montreal, Quebec, H4A 3T2 Canada; 6 Segal Cancer Proteomics Centre, Lady Davis Institute, Jewish General Hospital, McGill University, Montreal, Quebec, H3T 1E2 Canada; 7 Department of Neurology, Kawasaki Medical School, 577 Matsushima, Kurashiki, Okayama, 701-0192 Japan; 8 Department of Neuropediatrics and Muscle Disorders, Medical Center – University of Freiburg, Faculty of Medicine, Freiburg, Germany; 9 Centro Nacional de Análisis Genómico (CNAG-CRG), Center for Genomic Regulation, Barcelona Institute of Science and Technology (BIST), Barcelona, Catalonia, Spain; 10 Children’s Hospital of Eastern Ontario Research Institute, University of Ottawa, Ottawa, Canada and Division of Neurology, Department of Medicine, The Ottawa Hospital, Ottawa, Canada

**Keywords:** Caveolin-3, Caveolinopathy, LGMD1C, Chaperonopathy, Protein aggregate, Skeletal muscle proteomics

## Abstract

**Background:**

Caveolin-3 (CAV3) is a muscle-specific protein localized to the sarcolemma. It was suggested that CAV3 is involved in the connection between the extracellular matrix (ECM) and the cytoskeleton. Caveolinopathies often go along with increased CK levels indicative of sarcolemmal damage. So far, more than 40 dominant pathogenic mutations have been described leading to several phenotypes many of which are associated with a mis-localization of the mutant protein to the Golgi. Golgi retention and endoplasmic reticulum (ER) stress has been demonstrated for the CAV3 p.P104L mutation, but further downstream pathophysiological consequences remained elusive so far.

**Methods:**

We utilized a transgenic (p.P104L mutant) mouse model and performed proteomic profiling along with immunoprecipitation, immunofluorescence and immunoblot examinations (including examination of α-dystroglycan glycosylation), and morphological studies (electron and coherent anti-Stokes Raman scattering (CARS) microscopy) in a systematic investigation of molecular and subcellular events in p.P104L caveolinopathy.

**Results:**

Our electron and CARS microscopic as well as immunological studies revealed Golgi and ER proliferations along with a build-up of protein aggregates further characterized by immunoprecipitation and subsequent mass spectrometry. Molecular characterization these aggregates showed affection of mitochondrial and cytoskeletal proteins which accords with our ultra-structural findings. Additional global proteomic profiling revealed vulnerability of 120 proteins in diseased quadriceps muscle supporting our previous findings and providing more general insights into the underlying pathophysiology. Moreover, our data suggested that further DGC components are altered by the perturbed protein processing machinery but are not prone to form aggregates whereas other sarcolemmal proteins are ubiquitinated or bind to p62. Although the architecture of the ER and Golgi as organelles of protein glycosylation are altered, the glycosylation of α-dystroglycan presented unchanged.

**Conclusions:**

Our combined data classify the p.P104 caveolinopathy as an ER-Golgi disorder impairing proper protein processing and leading to aggregate formation pertaining proteins important for mitochondrial function, cytoskeleton, ECM remodeling and sarcolemmal integrity. Glycosylation of sarcolemmal proteins seems to be normal. The new pathophysiological insights might be of relevance for the development of therapeutic strategies for caveolinopathy patients targeting improved protein folding capacity.

**Electronic supplementary material:**

The online version of this article (10.1186/s13395-018-0173-y) contains supplementary material, which is available to authorized users.

## Background

**Fig. 1:**
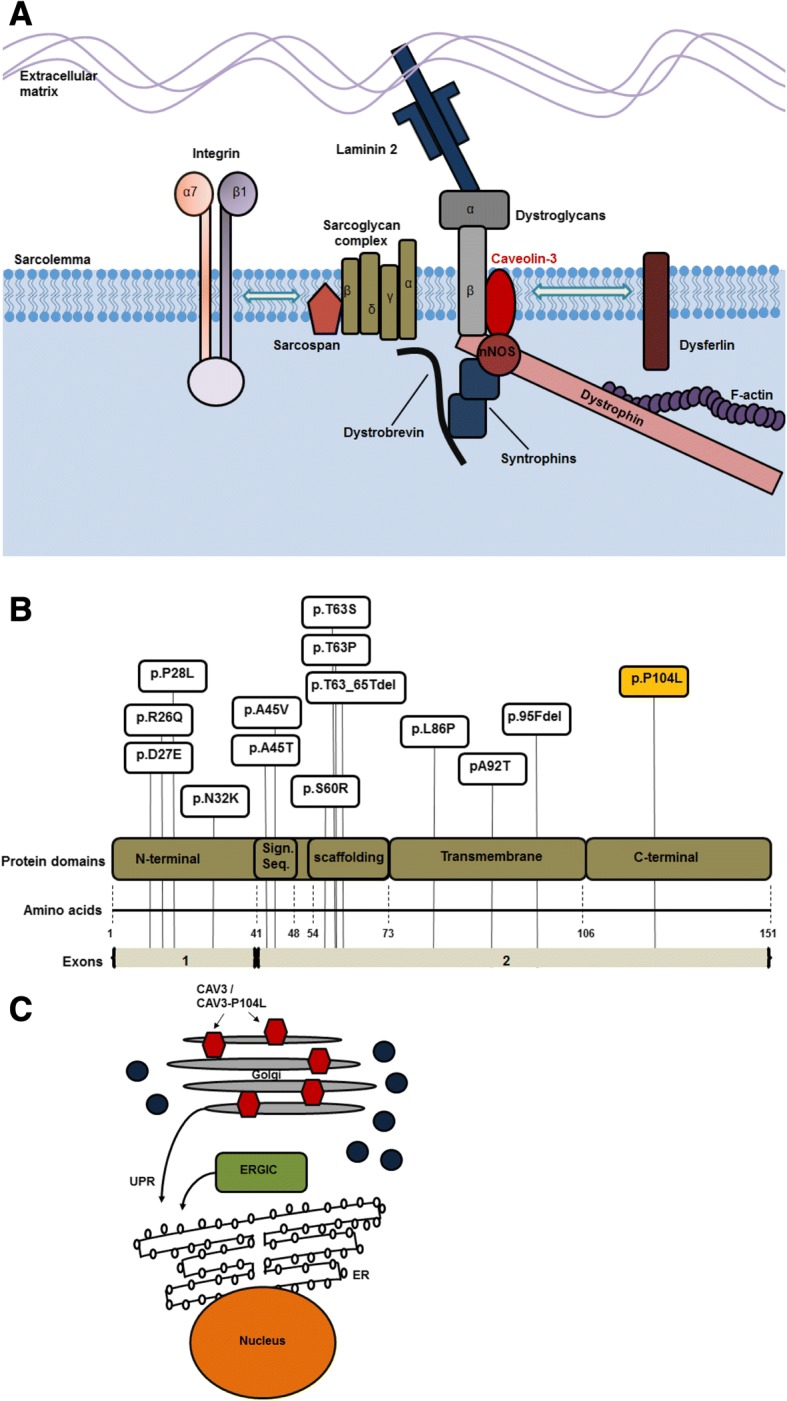
Muscle caveolinopathies are caused by mutations of the *CAV3* gene*.* **a** Subcellular localization of CAV3: the wild-type CAV3 protein localizes to the sarcolemma where it associates with components of the dystrophin-associated glycoprotein complex. CAV3 interacts directly with β-dystroglycan, nNOS and dysferlin. Moreover, CAV3 indirectly interacts with F-actin, syntrophin, dystrobrevin and laminin-2 as well as further components of the complex such as sarcoglycans and integrins. **b** Localization and distribution of paradigmatic CAV3 missense mutations leading to skeletal muscle phenotypes. The p.P104L mutant CAV3 protein is causative for Limb Girdle Muscular Dystrophy type 1 C. **c** Under physiological conditions, CAV3 is synthesized in the ER and transported through the Golgi to the sarcolemma. The missense mutant CAV3 proteins (hexagons in this carton) accumulate in the Golgi and cause ER stress and UPR activation

Caveolin-3 (CAV3), a muscle-specific member of the caveolin protein family, is a structural protein important for signal transduction, lipid metabolism, cell growth, mechanoprotection, autophagy, maintenance of neuromuscular junctions and apoptotic cell death [1–3]. CAV3 first appears during myoblast differentiation and is localized to the sarcolemma within caveolae, 50–100 nm flask-shaped invaginations of the plasma membrane. There, it associates with the dystroglycan complex establishing a connection between the extracellular matrix and the cytoskeleton (Fig. 1a). Muscle diseases caused by mutations in the *CAV3* gene are called caveolinopathies [1]. So far, more than 40 pathogenic *CAV3* mutations have been described which are localized within different protein domains (Fig. 1b) and are leading to different disease phenotypes including Limb Girdle Muscular dystrophy (LGMD), rippling muscle disease (RMD), distal myopathy (DM), hyperCKemia (HCK) and myalgia [1, 4]. A clear genotype-phenotype correlation does not exist, and some of the phenotypes may present as a clinical continuum [5–7]. However, dominant mutants are commonly associated with lowered sarcolemmal CAV3 levels. These are related to dissociation of the hetero-oligomers at the sarcolemma, degradation by the ubiquitin-proteasome pathway, and abnormal accumulation of mutated and wild-type (wt) CAV3 within the Golgi causing ER-stress and thus activation of the unfolded protein response (UPR) [8–10] (Fig. 1c).

A transgenic animal model expressing an additional copy of CAV3 harbouring the p.P104L mutation was published in 2001 by Sunada and co-workers [11]. Notoriously, this missense mutation has been identified in LGMD patients [1]. Proline residues in proteins, based on their biochemical properties, are important for structural maintenance of integral membrane proteins [12] such as CAV3. Hence, a substitution of proline to leucine in an integral membrane protein should be of pathogenic relevance. The phenotype of these mice has been examined extensively and appears to phenocopy the human disorder [9, 11, 13].

To better understand the consequences of CAV3 mutations, we chose the paradigmatic p.P104L missense mutation and investigated protein stability and aggregation, ER-Golgi pathology and the overall protein composition as well as glycosylation of the α-dystroglycan protein. For this purpose, quadriceps muscles of the above-mentioned animal model have been utilized and immunological, morphological and proteomic studies have been applied. Electron as well as coherent anti-Stokes Raman scattering (CARS) microscopic studies revealed presence of protein aggregates. Mass spectrometry-based characterization of these aggregates suggested mitochondrial, cytoskeletal, sarcolemmal and ECM vulnerability in p.P104L CAV3 diseased muscle. This molecular observation especially accords with mitochondrial perturbations detected on the ultra-structural and global proteomic level. Moreover, data of our global proteomic profiling revealed an increase of 77 and a decrease of 43 proteins. Notably, further immunological studies confirmed the proteomic findings. Interestingly, localization studies of sarcolemmal proteins—including components of the DGC—classified those as substrates of impaired ER-Golgi function. However, glycosylation of α-dystroglycan seems to be not impaired by the p.P104L CAV3 mutation caused altered ER-Golgi morphology.

## Methods

### Animals

The transgenic mouse model expressing p.P104L CAV3 was kindly provided by co-author Professor Yoshihide Sunada (Department of Neurology, Kawasaki Medical School, Okayama, Japan). Genotyping was performed as described previously [9, 11, 13]. All procedures were approved by the Uniklinik RWTH Aachen Institutional Animal Care and Use authorities. To obtain animals carrying the dominant p.P104L CAV3 mutation (Tg/+) as well as respective wild-type littermates (+/+), breeding of Tg/+ with +/+ was carried out.

### Light microscopy, immunohistochemistry and immunofluorescence

Five-micrometre sections were cut from formalin-fixed-paraffin-embedded (FFPE) muscle biopsy tissue. These were used for hematoxylin and eosin (H&E) staining and for immunohistochemistry (IHC) as well as for immunofluorescence (IF). For IHC and IF, sections were placed on silan-coated slides, treated with descending alcohol series for re-hydration and unmasked by heat (steam oven, citrate buffer). Afterwards, sections were blocked with 2% goat serum in PBS followed by incubation with primary antibodies (Additional file 1: Table S2) overnight. For IHC, peroxidase-labeled secondary antiserum (1:200, DCS, Hamburg, Germany) and diaminobenzodine (DAKO, USA) were used to detect antibody binding and cellular structures were counterstained with hematoxylin. For IF, Alexa 488- and Alexa 555-conjugated secondary antibodies (diluted 1:500, in 2% goat serum, respectively) were used to detect binding of primary antibodies and incubated at room temperature for 2 h followed by three washing steps in 1× PBS.

To systematically study Golgi-dispersion, length to width ratios have been calculated for 10 Golgi-structures (visualized utilizing a golgin-97 antibody) in type 1 fibres from three p.P104L CAV3 transgenic and wild-type animals aged 6 months and 1 year, respectively.

### Electron microscopic studies

Electron microscopic using glutaraldehyde-fixed, resin-embedded quadriceps muscles of 26 weeks old wt and mutant mice were performed as described previously [14].

### Coherent anti-Stokes Raman scattering microscopy

To obtain further information regarding the build-up of protein deposits, we employed—in addition to confocal fluorescence microscopy—coherent anti-Stokes Raman scattering (CARS). CARS is a non-linear variant of spontaneous Raman scattering and as such directly assesses the molecular vibrations of the sample itself. Therefore, CARS is inherently label-free and not limited by the availability of dyes or antibodies. A widespread use of CARS is its application in lipid biology [15]. In this application, the localization of lipids is examined at a specific wavenumber (2845 cm^−1^). By monitoring other wave lengths (= molecular vibrations), other targets can be investigated. Here, we focused on the spatial distribution of proteins at 2932 cm^−1^ [16].

CARS measurements were performed on a modified Leica TCS SP 8 CARS microscope with an APE picoEmerald as laser source. The 1064 nm output of the picoEmerald was used as Stokes, the OPO signal was tuned to 811 nm and provided both pump and probe. This combination results in a CARS signal at 655 nm and corresponds to a wavenumber of 2932 cm^−1^. Both lasers were fixed at a power of 900 mW at the picoEmerald output port. Further subsequent laser attenuation in the Leica systems was varied between 30 and 40%, depending on the sample. The laser beams were focused onto the sample using a Leica HCL IRAPO 40×/1.1 water or Leica HC PL APO 20×/0.75 CS2 objective. The resulting CARS signal was collected via the same objective in EPI (backward) direction and subsequently detected in the spectral regime of 560–750 nm via a photo multiplier tube (PMT) detector. The Second Harmonic Generation and Two-Photon fluorescence signals were collected at the same time (380–560 nm). Furthermore, all signals were also measured in a forward direction via a NA 0.55 condenser and the same filter/detector arrangement as in the EPI direction.

After the de-paraffinization step, the samples (quadriceps muscles from 6 weeks and 26 weeks old wild-type and p.P104L CAV3-transgenic animals, respectively) were thoroughly dried with a constant flow of dry air, measured in the CARS system. Afterwards, the staining protocol was pursued.

### Protein lysate preparation and immunoblot studies

Muscle tissue was transferred to muscle lysis buffer (0.125 M Tris, pH 6.4, 4% SDS, 10% β-mercaptoethanol, 10% glycerin, 0.001% bromophenol blue, 4 M urea and protease inhibitor mix) and lysed by sonication. Samples were chilled on ice and heated for 15 min at 56 °C. Protein concentration was quantified by BCA protein assay-reducing agent compatible kit (Pierce) according to the manufacturer’s instructions.

For immunoblot studies, 10 μg protein was used in each case, loaded on 10% polyacrylamide gels and separated for 120 min at 120 V. Following the separation, proteins were transferred to Immobilion-P PVDF membrane (0.45 μm, Millipore) over night at 10 V by tank-blot technique. Membranes were blocked with 1% casein buffer [1% casein; (Roche) in Tris-buffered saline (TBS) and 0.1% Tween20 (Sigma) as a 1:1 mix with maleic acid buffer composed of 100 mM maleic acid (Sigma) and 150 mM NaCl] for 2 h followed by four washing steps using TBS with 0.1% Tween20 (TBS-T). Membranes were incubated with several primary antibodies (Additional file 1: Table S2) at 4 °C (overnight) and then washed in TBS-T thrice. Horseradish peroxidase conjugated secondary goat anti-rabbit antibody (Sigma) or goat anti-mouse antibody (Sigma) was diluted at 1:25,000 and added to membranes for 1 h. In the following step, membranes were washed three times in TBS-T for 10 min. By using the enhanced chemiluminiscence, horseradish peroxidase substrate (Super-Signal West Pico and Super-Signal West Femto; Pierce) signals were detected on CL-X Posure films (Thermo Scientific).

For the study of α-dystroglycan glycosylation, wild-type and p.P104L mutant muscles were minced on ice and homogenized in 1% Triton X-100 TBS and protease inhibitors using the Bullet Blender (Next Advance, Averill Park, NY). Samples were enriched for glycoproteins using WGA agarose beads (Vector Labs) as described previously [17]. Protein levels in samples were measured using the DC Protein Assay (Bio-Rad, Hercules, CA), and equal amounts were added to wheat-germ agglutinin (WGA) agarose beads (Vector Labs, Burlingame, CA). Samples were then run out on a 3–15% gradient SDS-PAGE gel, after which the protein was transferred to PVDF membranes. The latter were blotted with the antibodies monoclonal IIH6-antibody, which recognizes specifically glycosylated DG, and the polyclonal antibody AF6868, which recognizes both α- and β-dystroglycan, as published previously [18]. Images were captured using the Licor (Lincoln, NE) system and Odyssey software.

### Global proteomic profiling study

#### Materials

The following materials were purchased from Sigma-Aldrich, Steinheim, Germany: ammonium hydrogen carbonate (NH_4_HCO_3_), guanidine hydrochloride (GuHCl), iodoacetamide (IAA) and urea. Tris base was obtained from Applichem Biochemica, Darmstadt, Germany. Sodium dodecyl sulfate (SDS) was purchased from Carl Roth, Karlsruhe, Germany. Dithiothreitol (DTT), EDTA-free protease inhibitor (Complete Mini) tablets were bought from Roche Diagnostics, Mannheim, Germany. Sodium chloride (NaCl) and calcium chloride (CaCl_2_) were from Merck, Darmstadt. Sequencing grade modified trypsin was from Promega, Madison, WI USA. Bicinchoninic acid assay (BCA) kit was acquired from Thermo Fisher Scientific, Dreieich, Germany. All chemicals for ultra-pure HPLC solvents such as formic acid (FA), trifluoroacetic acid (TFA) and acetonitrile (ACN) were obtained from Biosolve, Valkenswaard, The Netherlands.

#### Tissue lysis and carbamidomethylation

Six quadriceps muscles, i.e. three derived from 10 weeks old p.P104L CAV3-transgenic animals and three from wild-type littermates were collected, snap-frozen in liquid nitrogen at − 80 °C and processed independently. The whole quadriceps muscle was lysed in 1.0 mL of 50 mM Tris-HCl (pH 7.8) buffer containing 150 mM NaCl, 1% SDS, and Complete Mini. Tissue was homogenized by mechanical grinding followed by sonication with 1 to 1 s pulses (10 s) followed by centrifugation at 4 °C and 1600 g for 15 min. Protein concentration of the supernatant was determined by BCA assay according to the manufacturer’s protocol. Disulfide bonds were reduced by addition of 10 mM DTT at 56 °C for 30 min, and free sulfhydryl bonds were alkylated with 30 mM IAA at room temperature (RT) in the dark for 30 min.

#### Sample preparation and trypsin digestion

After filter-aided sample preparation (FASP) [19, 20], cell lysates corresponding to 100 μg of protein were diluted 10-fold with freshly prepared 8 M urea/100 mM Tris-HCl (pH 8.5) buffer and placed on a Microcon centrifugal device (30 kDa cutoff). The device was centrifuged at 13,500 g at RT for 15 min. All subsequent centrifugation steps were performed under the same conditions. To eliminate residual SDS, three washing steps were carried out with 100 μL of 8 M urea/100 mM Tris-HCl (pH 8.5). To exchange the buffer, the filter was washed thrice with 100 μL of 50 mM NH_4_HCO_3_ (pH 7.8). Then, proteins were incubated at 37 °C for 14 h with 100 μL of proteolysis buffer: trypsin (Promega) (1:25 *w*/*w*, protease to substrate), 0.2 M GuHCl and 2 mM CaCl_2_ in 50 mM NH_4_HCO_3_ (pH 7.8). The resulting tryptic peptides were recovered by centrifugation with 50 μL of 50 mM NH_4_HCO_3_ followed by 50 μL of ultra-pure water. Finally, peptides were acidified by addition of 10% TFA (*v*/*v*) and digests were quality controlled as described previously [21].

#### LC-MS/MS analysis

One-microgramme peptides of three mutant and three wild-type quadriceps muscles were analysed using an Orbitrap Elite mass spectrometer coupled with an Ultimate 3000 nano RSLC system. In all measurements, samples were analysed in randomized order to minimize systematic errors. Peptides were pre-concentrated on a 100 μm × 2 cm C18 trapping column for 10 min using 0.1% TFA (*v*/*v*) at a flow rate of 20 μL/min followed by separation on a 75 μm × 50 cm C18 main column (both Pepmap, Thermo Scientific) with (1) a 210-min LC gradient ranging from 3 to 42% of 84% ACN, 0.1% FA (*v*/*v*) at a flow rate of 230 nL/min. All samples were measured in a data-dependent acquisition.

In the OrbiElite, MS survey scans were acquired in the Orbitrap from m/z 300 to 1500 at a resolution of 60,000 using the polysiloxane ion at m/z 371.101236 as lock mass [22]. The ten most intense signals were subjected to collision-induced dissociation (CID) in the ion trap, taking into account a dynamic exclusion of 30 s. CID spectra were acquired with normalized collision energy of 35%. AGC target values were set to 106 for MS1 and 104 for ion trap MS2 scans, and maximum injection times were set to 100 ms for both full MS and MS2 scans.

#### Label-free data analysis

Data analysis of the acquired label-free quantitative MS was performed using the Progenesis LC-MS software from Nonlinear Dynamics (Newcastle upon Tyne, UK). For all measurements, biological triplicates of quadriceps muscle were compared to the corresponding control triplicates separately.

The MS raw data was aligned by Progenesis which automatically selected one of the MS files as reference. After peak picking, only features within retention time and m/z windows from 0 to 200 for OrbiElite data and 300–1500 m/z, with charge states + 2, + 3, and + 4 were considered for peptide statistics, analysis of variance (ANOVA) and principal component analysis (PCA). The MS/MS spectra were exported as peak lists. The lists were then searched against a concatenated target/decoy version of the mouse Uniprot database, (downloaded on 11th of December 2013, containing 20,273 target sequences) using Mascot 2.4 (Matrix Science), MS-GF+, and X!Tandem Jackhammer (2013.06.15) with the help of searchGUI 1.24.0 [23]. As a proteolytic enzyme, trypsin was selected with a maximum of two missed cleavages. Carbamidomethylation of Cys was set as fixed modification and oxidation of Met was selected as variable modification. MS and MS/MS tolerances were set to 10 ppm and 0.5 Da, respectively. Combined search results were filtered at a false discovery rate (FDR) of 1% on the protein level and exported using the PeptideShaker [24] software 0.28.0 (http://compomics.github.io/projects/peptide-shaker.html) features which allows a direct re-import of the quality-controlled data into Progenesis. Peptide sequences containing oxidized Met and pyro-Glu (derived from X!Tandem 2nd pass search) were excluded for further analysis. Only proteins that were quantified with unique peptides were exported. Then, for each protein, the average of the normalized abundance (obtained from Progenesis) from the triplicate analyses was calculated to determine the ratios between the controls and transgenic muscle samples. Only proteins matching the following criteria were considered as regulated: they had to be (i) commonly quantified in all the replicates with (ii) an ANOVA *p* value of < 0.05 (Progenesis) and (iii) an average ratio < 1.24 or > 0.84—all parameters depending on data distribution. Proteins identified with at least two unique peptides were classified as confidential class I and proteins identified with solely unique peptide were classified as confidential class II.

### Immunoprecipitation of protein aggregates and mass spectrometry-based analysis

#### Materials

See above.

#### Sample preparation, cleanup and tryptic digestion

To further characterize the protein aggregates detected via electron and CARS microscopy as well as via immunofluorescence studies on a molecular level, utilizing antibodies targeting ubiquitin (ab7780; Abcam) and p62/SQSTM1 (ab109012; Abcam) (Additional file 1: Table S2) ubiquitinated as well as proteins binding to p62 were precipitated from muscle (5 mg starting material) derived from four 26 weeks old p.P104L CAV3-transgenic and wild-type animals respectively utilizing the “Pierce Protein A/G Magnetic Beads” kit (Thermo Fisher Scientific). Precipitations were performed according to the manufacturers’ instructions. To later exclude proteins non-specifically binding to the magnetic beads, for p.P104L CAV3 mutant and wild-type samples (four respectively) whole protein extracts have been incubated with the beads without adding the antibodies. After elution, samples were subjected to ethanol precipitation, which was used as a cleanup method: protein solution was 10-fold diluted (ratio of 1:10) with ultrapure ice-cold ethanol (100%), and then stored at − 40 °C for 60 min followed by centrifugation at 4 °C for 30 min at 18,000 g. The pellet was dried under a laminar flow hood and then solubilized with a solution containing 1 M GuHCl and 50 mM NH_4_HCO_3_ (pH 7.8). Proteins were reduced and alkylated as described above. Next, samples were diluted with 50 mM NH_4_HCO_3_ (pH 7.8) until a concentration of 0.2 M GuHCl was attained. For the tryptic-digestion, the protein concentration was roughly estimated as being 5 μg. Protein cleavage was achieved by adding trypsin to the sample in a ratio of 1:20 (enzyme to substrate) followed by incubation for 12 h at 37 °C.

Clean-up of tryptic peptides was performed with commercially available C18 tips (Omix). The tips were firstly activated by using 100% ACN followed by equilibration with 0.1% TFA. All volumes used were assessed based on the size/capacity/type of tips. After equilibration of the resin, the peptide mixture was loaded onto the tips and the flow through passed two times and was then discarded. Afterwards, the column was washed three times with 0.1 %TFA to ensure proper elimination of contaminants. Finally, peptides were eluted from the resin with 60% ACN in 0.1 %TFA. The eluates were dried in the SpeedVac and then resuspended in 0.1% TFA.

#### LC-MS/MS analysis

The samples were analysed using a Velos ion trap mass spectrometer coupled with an Ultimate 3000 RSLC system (both Thermo Scientific). Peptides were separated as described above using a 90-min LC gradient ranging from 3 to 42% of 84% ACN, 0.1% FA (*v*/*v*) at a flow rate of 300 nL/min. The full MS scans were acquired in the m/z 300 to 2000 at a resolution of 60,000 using the polysiloxane ion at m/z 371.101236 as lock mass. The ten top most intense ions were subjected to collision induced dissociation (CID) with an NCE of 35% in the ion trap, considering a dynamic exclusion of 30 s. AGC target values were set to 10^6^ for MS1 and 10^4^ for ion trap MS2 scans, and maximum injection times were set to 100 ms for both full MS and MS^2^ scans.

#### Data analysis

Raw files were analysed in Proteome Discoverer 2.2.0388 (Thermo Scientific). Searches were conducted in a target/decoy manner against a concatenated protein sequence database (*Mus musculus*, 16,966 target sequences, downloaded from UniProt in October 2017 and a common contaminant database comprising 247 target sequences). Samples were searched using Mascot 2.6.1 (Matrix Science) and the precursor mass tolerance and fragment mass tolerance were set to 10 ppm and 0.02 Da, respectively. For all searches, enzyme specificity was set to fully tryptic with an allowed maximum number of two missed cleavages. Carbamidomethylation of Cys (+ 57.02146 Da) was defined as fixed, while oxidation of Met (+ 15.99491 Da) and acetylation of protein N-termini (+ 42.01056 Da) were allowed as variable modifications. For the ubiquitin IP samples diGly was set as a variable modification as in this case the tryptic digest contains a diglycine-modified lysine, which serves as a signature for ubiquitination. False discovery rate (FDR) estimation was performed by the percolator node and all data were filtered to meet a 1% FDR-level for PSMs as well as peptides. Resulting dataset were further filtered for 1% FDR on the protein level, additionally excluding proteins without uniquely assigned peptides and such not marked as ‘master’ proteins. All proteins non-specifically binding to the magnetic beads were excluded.

### Transcript studies

RNA was extracted from quadriceps muscles derived from 26-week-old p.P104L CAV3 transgenic and control animals (RNeasy®, Qiagen). Total RNA was reverse transcribed using Superscript II reverse transcriptase (Invitrogen). Quantitative real-time PCR was performed using the C1000 Thermal Cycler CFX96 (BioRad). For cDNA amplification of *dysferlin* (*Dysf*), *caveolin-3* (*Cav3*), *integrin-β-4* (*Igtb4*), *δ-sarcoglycan* (*Scgd*) and *glyceraldehyde-3-phosphate dehydrogenase* (*Gapdh*), a reaction mix containing Sso Fast Eva Green Supermix (BioRad) was used in combination with the following primers: *Gapdh* forward primer 5′ TGGCAAAGTGGAGATTGTTG 3′; *Gapdh* reverse primer 5′ CATTATCGGCCTTGACTGTG 3′; *Dysf* forward primer 5′ CTTACCACAGATGGACGATGC 3′; *Dysf* reverse primer 5′ CATAGAGGGAAACATCGCAGGC 3′; *Cav3* forward primer 5′ GACATTGTGAAGGTAGATTTTG 3′; *Cav3* reverse primer 5′ GGTAGCTCTTAATGCAGGGC 3′; *Igtb4* forward primer 5′ CTACTATGAGAAGCTCCATAAG 3′; *Igtb4* reverse primer 5′ CATTGTATGTGCCCACTTCCC 3′; *Scgd* forward primer 5′ GCTGGTGACAGGTCCGAAGGC 3′; *Scgd* reverse primer 5′ GTGCCTTCAGCTCCTAAGACTC 3′. Data were normalized with *glyceraldehyde-3-phosphate dehydrogenase* (*Gapdh*) as internal standard using the 2–ΔΔC T method [28].

## Results

### Mutant Caveolin-3 (p.P104L) protein is less stable than wild-type

**Fig. 2:**
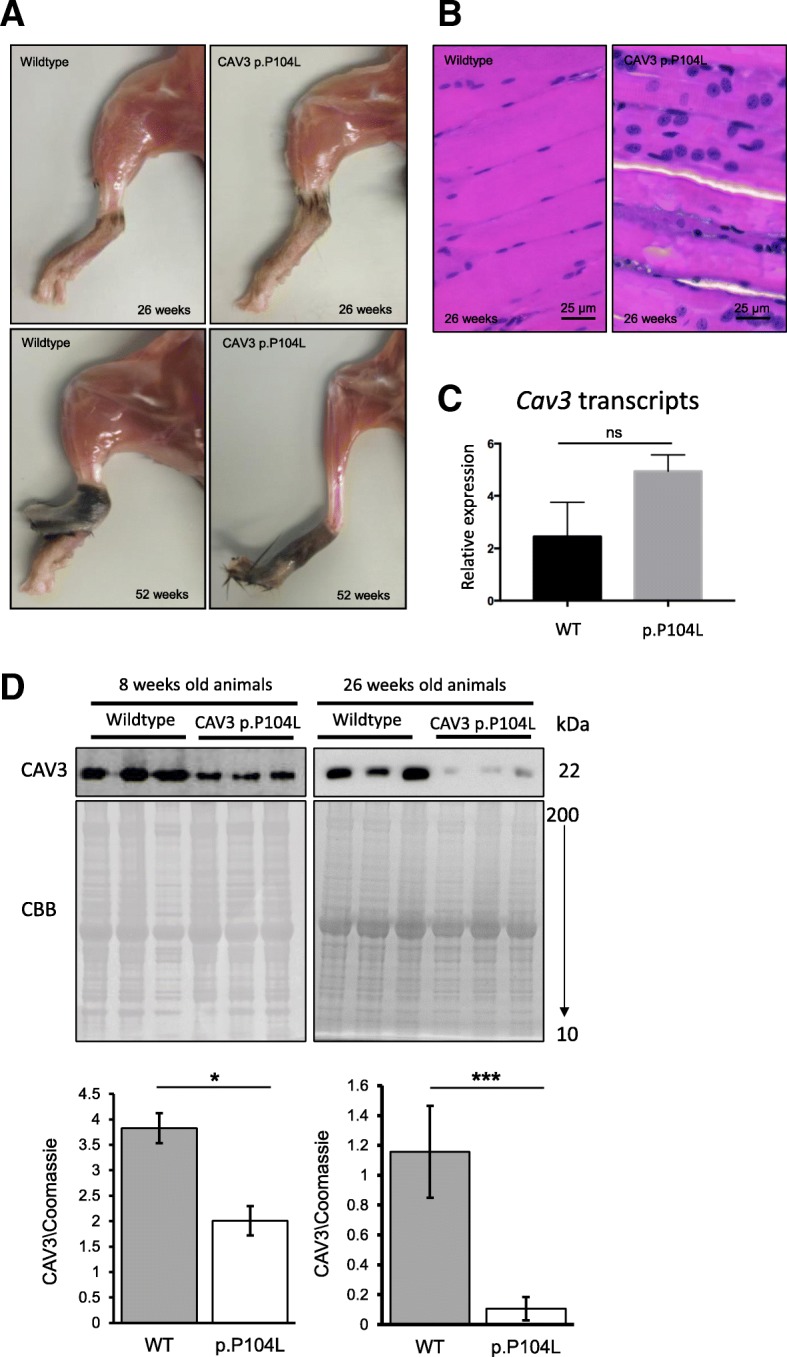
The CAV3 p.P104L mutation causes muscle pathology and influences protein stability. (**a**) Macroscopic comparison of hind limbs at 26 weeks wildtype mouse and a CAV3 (p.P104L) transgenic mouse reveals a moderate decline in muscle mass in the transgenic mouse which more pronounced at 52 weeks. (**b**) H&E staining reveals a largely normal appearance of wildtype quadriceps muscle at age 26 weeks compared to the quadriceps muscle of a transgenic animal presenting with muscle fiber caliber variability and internalized muscle fiber nuclei (Paraffin sections) (**c**) Transcript studies of Cav3 in quadriceps muscle of 26 weeks old p.P104L CAV3 mutant animals and wildtype littermates revealed increased cDNA abundance p.P104L CAV3 mutants. (**d**) Immunoblot of quadriceps muscle protein extracts (8 and 26 weeks old animals) revealing a prominent statistically significant CAV3 decrease in the mutant muscle. Coomassie blue staining was used as loading control

In caveolinopathy, mutant muscle fibres are known to present with absence of sarcolemmal CAV3 (drastically reduced immunoreactivity). This pathological observation is often associated with the presence of small dot-like CAV3-immunoreactive deposits scattered in the sarcoplasm [11]. Prompted by these known pathophysiological findings, transcript and protein studies of p.P104L CAV3 have been carried out utilizing quadriceps muscle derived from transgenic animals (Fig. 2a–c): results of our *CAV3* transcript studies revealed increased abundances in quadriceps muscle of p.P104L CAV3 mutant animals compared to wild-type littermates (both 26 weeks of age; Fig. 2c). Immunoblot studies of CAV3 quadriceps muscles from 6 and 26 weeks old transgenic animals compared to the respective wild-type littermates confirmed lowered abundance of the protein (Fig. 2d), suggesting a reduced stability of the mutant-protein (formation of irregular CAV3 wild-type-mutant protein complexes which undergo proteolysis). Interestingly, level of the CAV3 wild-type-mutant protein complex seem to decline with disease progression suggesting forced degradation (Fig. 2d). To exclude a direct impact of the p.P104L mutation on CAV3 antigene detection, a rabbit polyclonal peptide antibody corresponding to mouse caveolin-3 amino acids 1 to 19 (ab2912; Abcam) was utilized. However, our combined results supporting the concept of reduced protein stability rather than reduced stability of the mutant transcript.

### p.P104L Caveolin-3 causes Golgi/ER stress

Mis-localization of CAV3 aggregates (formed by mutant and wild-type protein) to the Golgi has already been described [8]. This pathological finding is also in line with the previous description of UPR activation [9] which in turn suggests a more generalized stress burden of the secretory apparatus comprised by discrete paired Golgi stacks in close proximity to exit sites from the ER (Fig. 1c). Here, we further investigated the presence of Golgi/ER stress by studying the structure of the secretory apparatus on the morphological level and UPR-related as well as Golgi-ER-shape-related factors on the protein level.

**Fig. 3:**
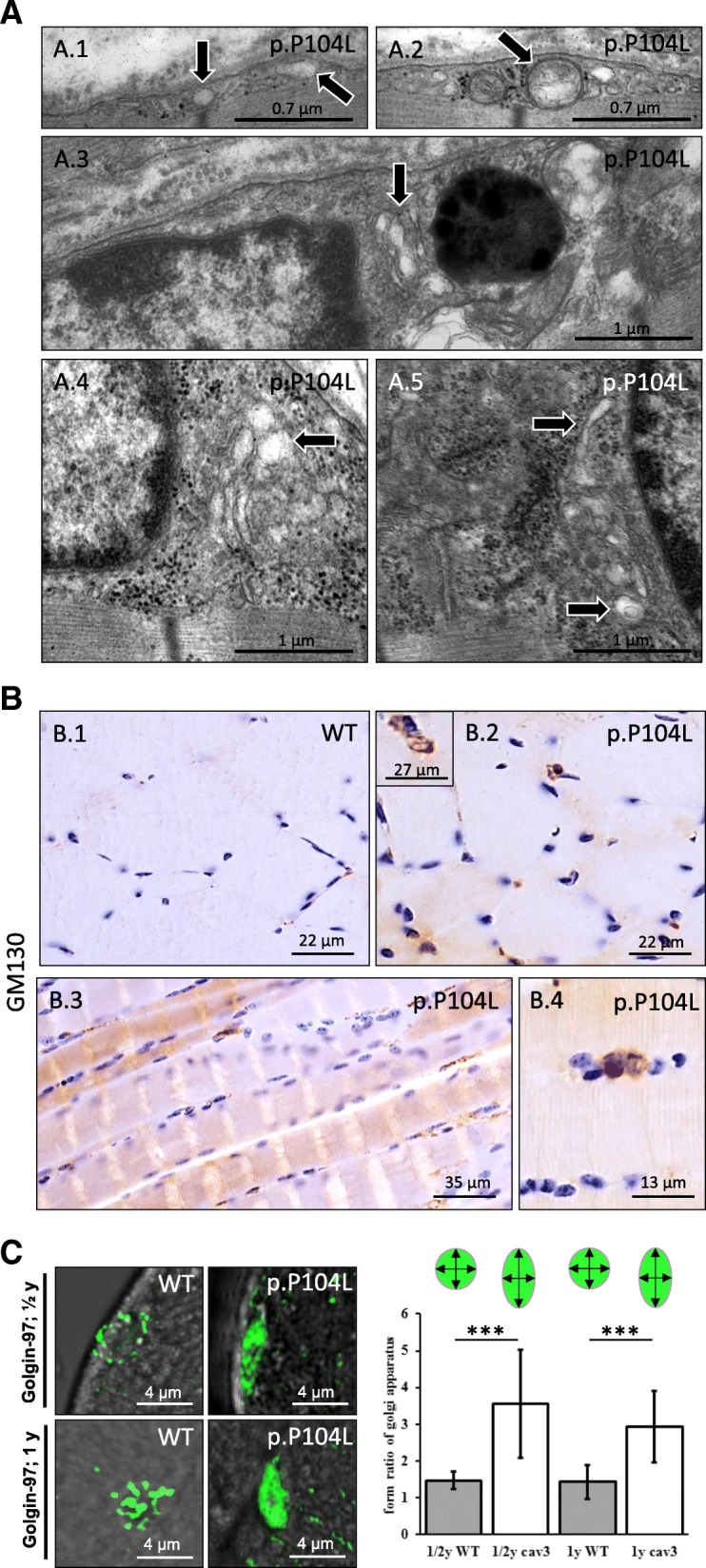
Expression of p.P104L missense mutant CAV3 disturbs ER-Golgi integrity. (**a**) EM findings in p.P104L CAV3 mutant quadriceps muscle: subsarcolemmal accumulation of vesicular structures (most likely corresponding to abnormal caveolae) (black arrows in 3A.1 and 3A.2) and dispersed ERGIC-Golgi structures (black arrows in 3A.3 – 3A.5). (**b**) Immunohistochemical studies focusing on ER-Golgi stress confirm GM130 increase. (**c**) Immunofluorescence studies focusing on Golgi-structure in 6 month and 1 year old animals (visualization via golgin-97 staining) revealed a statistically significant dispersion on p.P104L CAV3 transgenic animals

**Fig. 4:**
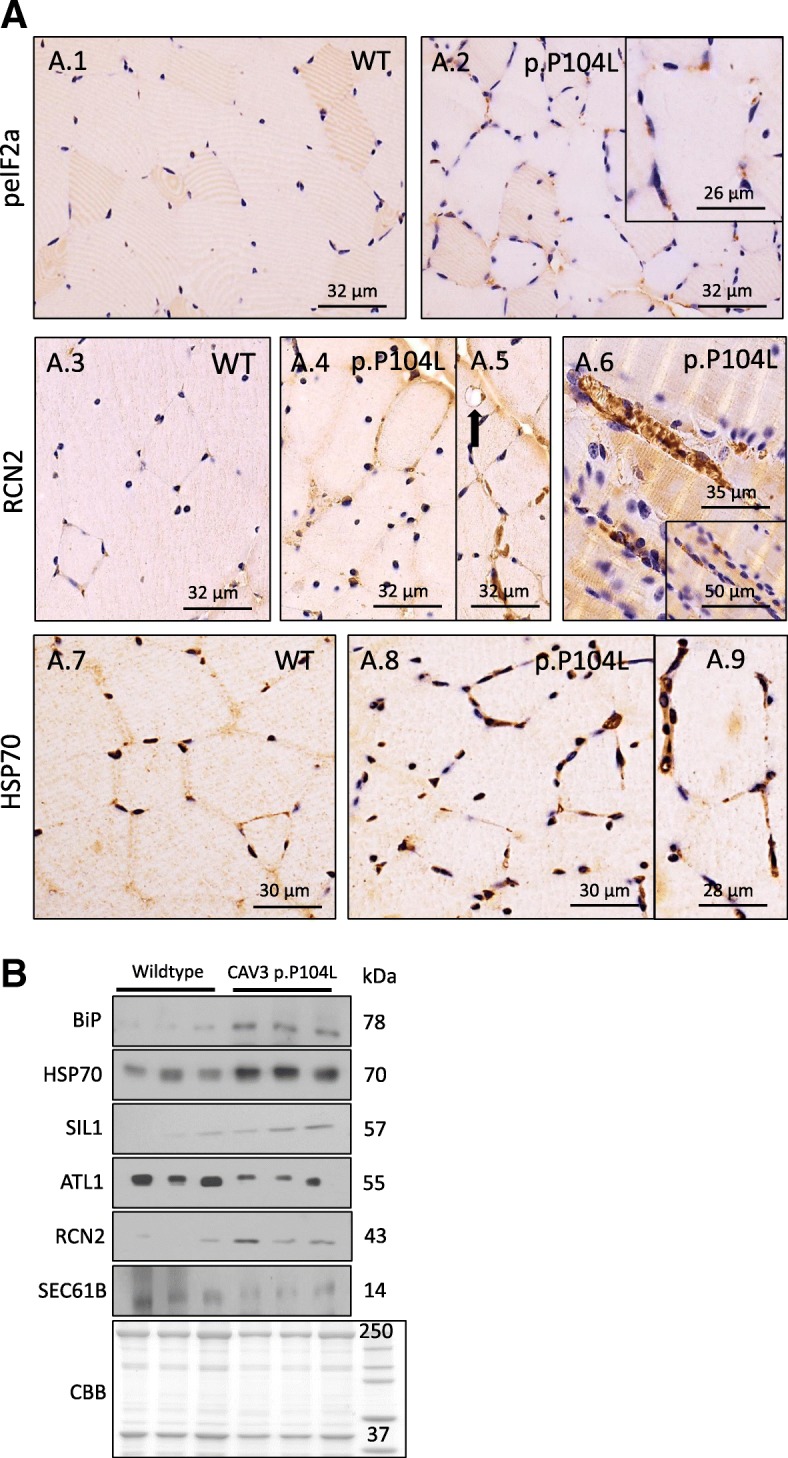
Study of ER-Golgi relevant proteins. (**a**) Immunohistochemical studies focusing on ER-Golgi stress confirm UPR activation (increased abundance of the phosphorylated (activated) form of eIF2α, of HSP70 and RCN2) in p.P104L muscle fibres. Interestingly, the cytosolic chaperone HSP70 shows enrichment at the sub-sarcolemmal sarcoplasm of muscle fibres expressing the mutant protein. RCN2 is accumulating in damaged muscle fibres. (**b**) Pathomorphological findings highlighted in Figure 3 are accompanied by reduced expression of ATL1, a protein important for the structural maintenance of the ER-Golgi system as well as altered abundance of proteins controlled by ER-stress and UPR (SIL1 and BiP, HSP70, RCN2 and SEC61). Coomassie brilliant blue staining: loading control

EM of muscles derived from 26-week-old mutant animals confirmed that membrane-bound vacuoles accumulate sub-sarcolemmally (Fig. 3 A.1, A.2). These vacuoles most likely correspond to abnormal caveolae and/sarcoplasmic reticulum cisternae [10]. In addition, we found widened and proliferated ERGIC-Golgi structures (Fig. 3 A.3–A.5). These ultrastructural findings correlate with increased and dispersed distribution of GM130 (Fig. 3 B.1–B.4), a peripheral membrane component of the cis-Golgi stack. GM130 acts as a membrane skeleton maintaining the structure of the Golgi apparatus, and as a vesicle tether that facilitates vesicle fusion to the Golgi membrane [25]. Golgin-97 immunoreactivity was also analysed by fluorescence microscopy and demonstrated pathological dispersion of Golgi structures in muscle fibres of p.P104L CAV3 transgenic animals (Fig. 3c). Decreased abundance of Atlastin-1 (Fig. 4b) was in line with Golgi pathology as this protein mediates homotypic fusion of ER membranes and functions in ER tubular network biogenesis and in Golgi biogenesis.

Correspondingly, immunoblots and immunohistochemistry revealed forced phosphorylation of eIF2α and increased abundance of the SR-resident SIL1-BiP chaperone complex and of HSP70 (sarcoplasmic chaperone stabilizing proteins against aggregation) as well as decreased SEC61 levels in the muscle fibres of p.P104L CAV3 transgenic animals (Fig. 4a, b, Additional file 2: Figure S3C). The SEC61 protein is the major component of a channel-forming translocon complex that mediates co-translational translocation of nascent (poly) peptides across the ER membrane; its downregulation is in accordance with an attenuation of protein synthesis to reduce the protein cargo through the secretory apparatus [26]. Increased levels of RCN2, an ER-luminal protein involved in Ca^2+^ and redox homeostasis [27], further suggests vulnerability of proper ER-Golgi function based on p.P104L Caveolin-3 expression.

### p.P104L Caveolin-3 induced changes in protein composition in the quadriceps muscle

**Fig. 5:**
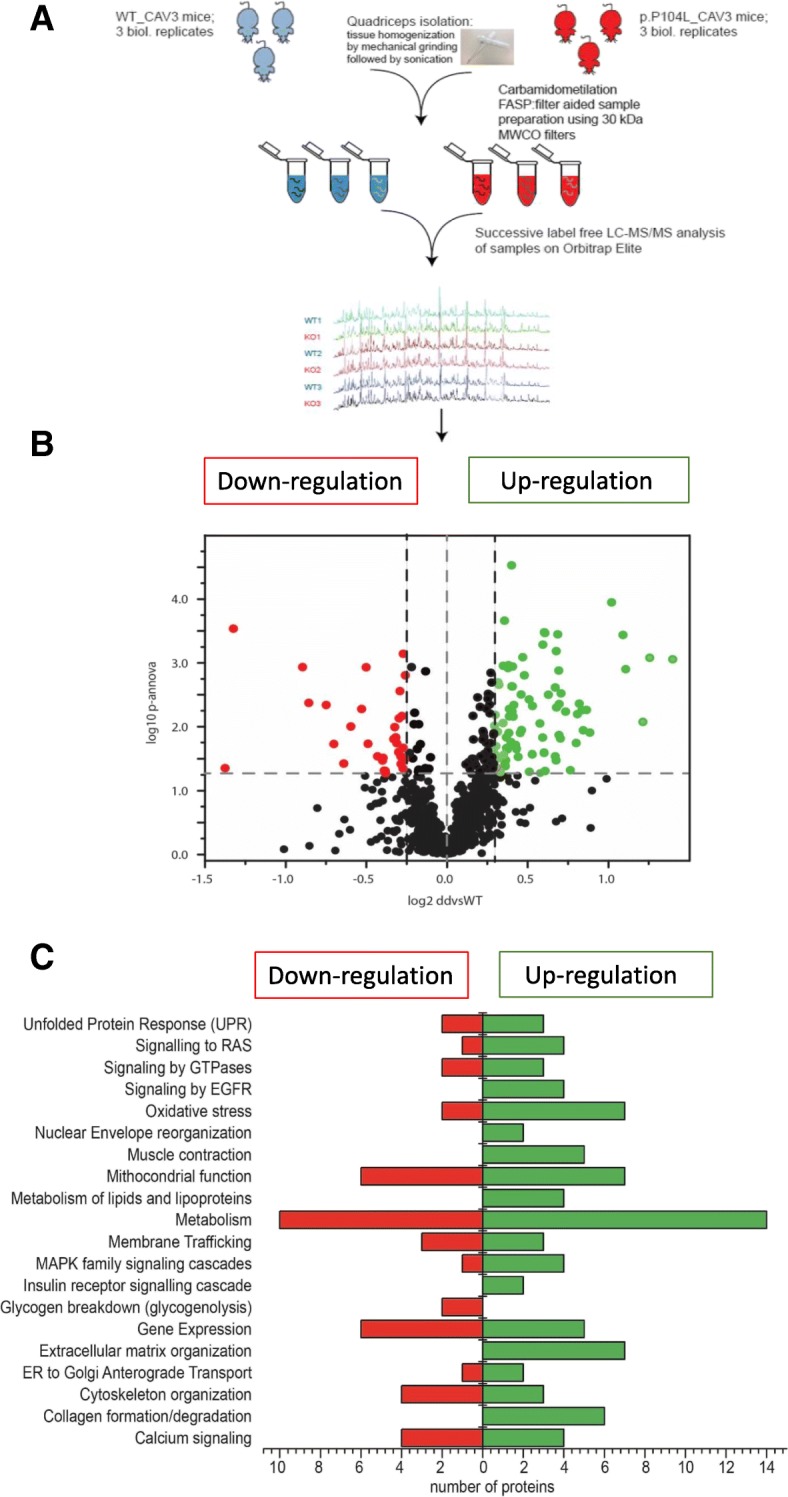
Proteomic studies to unravel the molecular nature of p.P104L induced muscular dystrophy in mice. **a** Proteomic workflow applied in this study. **b** Results of our label-free shotgun proteomic profiling are shown as a volcano plot. All points (each one represents a protein) over the horizontal line have a statistically significant *p*-ANOVA of ≤ 0.05. In total, 43 proteins are decreased (red points) and 77 proteins are increased in abundance (green points) in mutant mouse muscle. **c** Results of an in silico pathway analysis of proteins altered in abundance confirm alterations of the protein processing machinery, vulnerability of mitochondria as well as altered in ECM protein processing and cellular metabolism. In addition, there is evidence of oxidative stress and sarcolemmal vulnerability

Proteomic profiling is a useful approach to obtain unbiased insights into disease pathologies and related compensatory mechanisms (for example: [10, 29, 30]). We compared the proteomic signature of quadriceps muscles from three transgenic and wild-type littermate animals (age 10 weeks; muscles from the right legs of male animals). Using liquid chromatography coupled to tandem mass spectrometry (LC-MS/MS) for quantitative proteomics based on label-free protein quantification (Fig. 5a), we identified 77 proteins with increased and 43 proteins with decreased abundance (≥ 2 unique peptides, 1% FDR), respectively (Fig. 5b and Additional file 3: Table S1). Proteins localized to or processed in the Golgi-ER network (including proteins of the secretory pathway; ECM components), to the sarcolemma, to mitochondria, to the sarcoplasm (cytoskeleton), the myonucleus, and proteins shuttling between the two latter compartments were predominantly altered (Additional file 4: Figure S1 and Additional file 3: Table S1). Involvement of proteins localized to subcellular the ER-Golgi network accords with the morphological findings (Fig. 3). Several proteins showing altered expression are major determinants of skeletal muscle function (Additional file 3: Table S1), such as components of the dystrophin-associated glycoprotein complex (DGC), or have otherwise been linked to myopathic disorders directly; these include dysferlin, sarcoglycans, dystrophin and desmin (Additional file 3: Table S1). Using STRING software (https://string-db.org//), a functional interplay between the altered proteins could be demonstrated (Additional file 4: Figure S1) that might have been modified by the p.P104L-mutated CAV3. Pathway analysis suggests involvement of sarcolemma, protein processing and clearance machinery, mitochondria and extracellular matrix upon the presence of the dominant p.P104L CAV3 mutation (Fig. 5c and Additional file 5: Figure S2).

### p.P104L CAV3 leads to protein aggregate formation in quadriceps muscle fibres

**Fig. 6:**
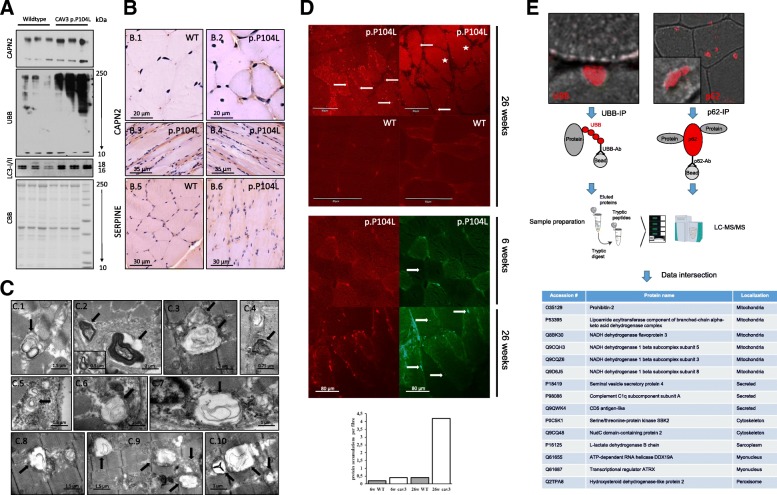
Activation of proteolysis and protein aggregation in p.P104L caveolinopathy. **A** Immunoblot studies confirming the increased abundance of CAPN2 identified in the proteome profile and showing increased abundance of ubiquitinated proteins as well as of LC3. Coomassie brilliant blue was used as loading control. **B** Increased abundance of CAPN2 was confirmed via immunohistochemical studies which also revealed elevated level of SERPINE (a protein functioning as a protease) at the sub-sarcolemmal region. **C** EM showing autophagic vacuoles containing electron-dense myelin-like structures (6C.1–6C.7) in quadriceps muscles of animals with advanced myopathy as well as damaged, probably degenerating mitochondria (6C.8–6C.10). **D** CARS (two photon) microscopy confirms the presence of sarcoplasmic and sub-sarcolemmal protein-dense structures in quadriceps muscle fibres of p.P104 CAV3 transgenic animals (red fluorescent dots) but not in control muscles from wild-type littermates. Concomitant second harmonic generation signals (green fluorescent dots) corresponding to organized molecular structures such as composed myosin and myelin also indicate the presence of small abnormal build-up of organized structures within the sarcoplasm of quadriceps muscle fibres of diseased animals frequently overlapping with the protein aggregates identified by two photon microscopy. Comparative analyses and quantification of aggregates in younger and older animals revealed that aggregate-build-up coincides with disease progression. **E** Immunoprecipitation of ubiquitinated and p62-binding proteins with subsequent mass spectrometry-based identification towards the molecular characterization of the protein aggregates detected via electron and CARS microscopy. Data intersection revealed similar alteration of 15 proteins

EM of muscles from p.P104L transgenic animals revealed perturbed ER-Golgi structures indicative for altered protein quality control. This is supported by immunoblot studies showing that several structural components of the ER-Golgi network are altered by the presence of the mutated protein. Moreover, proteomic findings show that proteins involved in protein quality control are altered in abundance, and several proteins involved in proteolysis present with altered abundance in p.P104L diseased muscle fibres (Additional file 4: Figure S1 and Additional file 3: Table S1). We hypothesized that prolonged ER-Golgi stress might lead to the build-up of protein aggregates and thus investigated quadriceps specimen derived from mice at a more advanced stage of caveolinopathy (26 weeks of age). Results of immunoblot and immunohistochemical/immunofluorescence studies confirmed the increased expression of CAPN, showed increase of LC3 (functions in autophagy substrate selection and autophagosome biogenesis) and revealed an elevation in overall ubiquitinated (UBB) proteins as well as sarcoplasmic p62-immunopositive deposits (Fig. 6a, b, e, Additional file 2: Figure S3C). These findings are compatible with the occurrence of myelin-like electron-dense material (indicative of autophagy) within the sarcoplasm and mitochondria (Fig. 6c) and with the increase of sarcoplasmic chaperones and proteins involved in protein breakdown (Fig. 4; Fig. 6a, b; Additional file 3: Table S1). In addition, our CARS-microscopic studies revealed the presence of sarcoplasmic and sub-sarcolemmal protein aggregates. Comparison of 6-week and 26-week-old transgenic and wild-type animals revealed that aggregate-build-up coincides with disease progression (Fig. 6d, upper panel and left images of lower panel as well as diagram).

Second harmonic generation signals (green) were collected at the same time and detect well-ordered structures such as collagen, composed myosin or myelin[31–33]. Notably, such structures detected in muscle fibres of the mutant animals frequently overlapped with the signals for the protein aggregates (red) (Fig. 6d, right images of lower panel), a finding which agrees with the presence of autophagic vacuoles detected by EM (Fig. 6c).

To identify substrates of protein aggregation, ubiquitinated and p62-binding proteins were precipitated and further analysed via mass spectrometry (Fig. 6e). Results of these studies allowed the identification of 37 ubiquitinated proteins in p.P104L CAV3 mutant muscle. Seven of these proteins represent cytoskeletal components or cytoskeleton modulating proteins, 6 proteins belong to mitochondria, 10 to myonuclei, 4 to sarcoplasm, 5 are regularly secreted, and 1 proteins belongs to peroxisomes, endosomes and the sarcolemma, respectively (Additional file 3: Table S1). Molecular characterization of p62 immunoprecipitates lead to the identification of 126 proteins binding to p62 in p.P104L CAV3 mutant muscle. Eighteen of these proteins represent cytoskeletal components or cytoskeleton modulating proteins, 26 proteins belong to mitochondria, 21 to myonuclei, 18 to sarcoplasm, and 10 are regularly secreted. Moreover, 9 proteins belong to the sarcolemma and the SR, respectively. Further 4 proteins are known components of the proteasome and 2 are normally localized to peroxisomes (Additional file 3: Table S1). Alteration of 9 proteins belonging to the SR accords with the vulnerability of the ER-Golgi system against expression of the p.P104L CAV3 mutant protein. However, intersection of the data of both independent immunoprecipitation experiments revealed 15 proteins ubiquitinated and binding to p62 in muscle of the caveolinopathy mouse model (Fig. 6e, Additional file 3: Table S1). Interestingly, 6 of those are mitochondrial proteins confirming the concept of mitochondrial vulnerability as already suggested by our electron microscopic and global proteomic findings. Further 3 of these proteins are regularly secreted, 2 belong to cytoskeleton, 2 to myonuclei and 1 protein normally localizes to the sarcoplasm and to peroxisomes, respectively.

### Components of the dystrophin-associated glycoprotein complex are substrates of the impaired protein quality control in p.P104L caveolinopathy

**Fig. 7:**
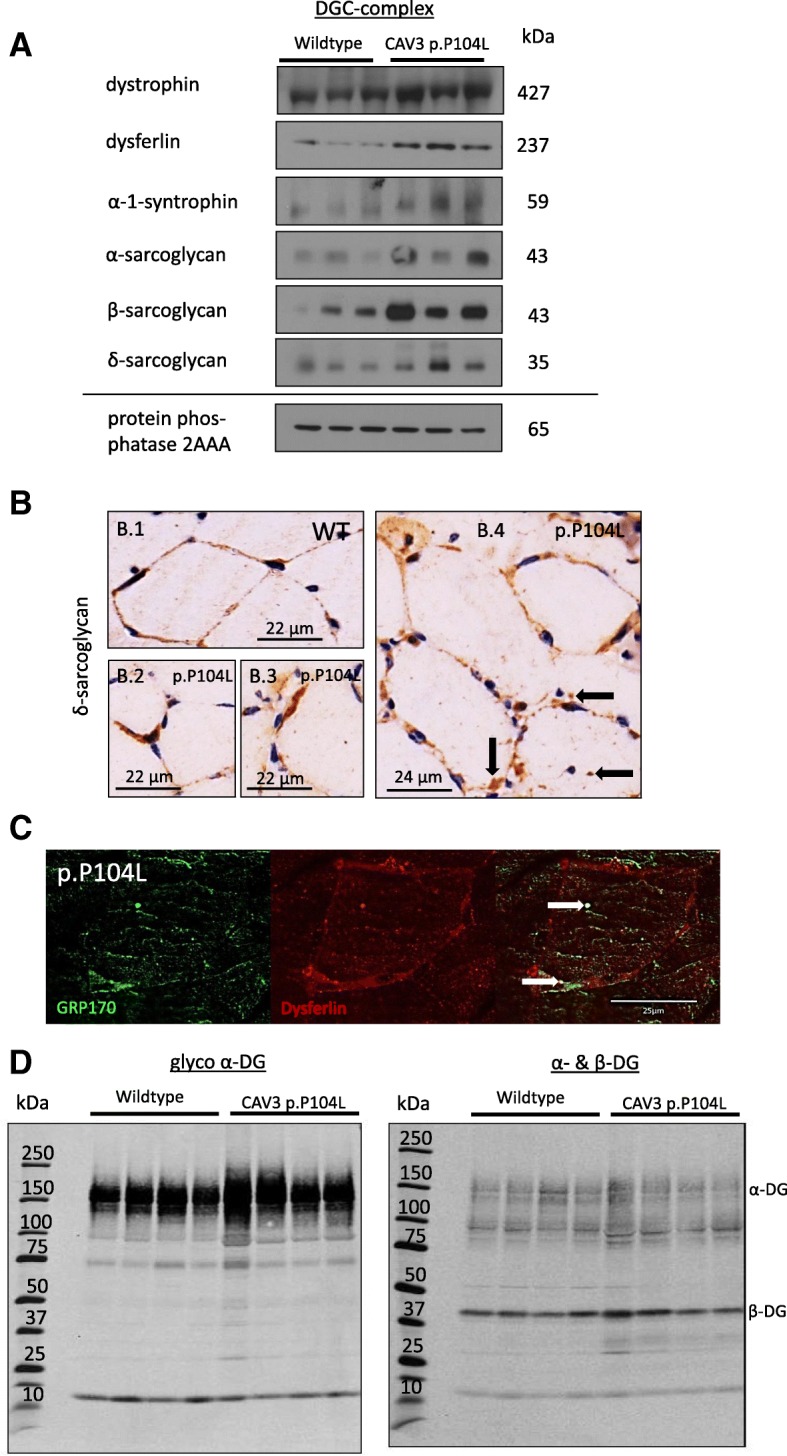
DGC components are substrates of the impaired protein processing machinery in p.P104L caveolinopathy. **A** Immunoblots of paradigmatic components of the DGC confirm the proteomic findings by showing increased protein abundance of dystrophin, dysferlin, α- and β-dystroglycan, α-1-syntrophin as well as α-, β- and δ-sarcoglycan. Protein phosphatase 2AAA, stable in our proteome profile, has been used as loading control, and shows stable levels, in line with our proteomic findings. **B** Increased abundance of DGC components was also confirmed by immunohistochenistry focusing on δ-sarcoglycan as a paradigmatic example. Interestingly, apart from occasional sarcoplasmic deposits (black arrows in 7B.4), an enrichment in the sub-sarcolemmal region could be identified (7B.2 and 7B.3). **C** Immunofluorescence-based co-localization studies showing (irregular) sarcoplasmic dots immunoreactive for GRP170 (a co-chaperon of the SIL1-BiP machinery) and dysferlin. **D** Immunoblot analysis utilizing the monoclonal antibody IIH6, which recognizes specifically glycosylated α-dystroglycan (left panel) and the polyclonal antibody AF6868, which recognizes both α- and β-dystroglycan (right panel)

As described above, our comparative proteome profiling revealed altered abundance of several DGC components (Additional file 3: Table S1). Based on the results of this unbiased screening, we hypothesized that proteins of the DGC might be affected indirectly by p.P104L mutant CAV3 mis-localization to the Golgi, ER-stress response activation and impaired protein quality control. Further immunoblot and immunohistochemical studies focussing on paradigmatic components of the complex confirmed the increase of the same proteins detected via shotgun proteomics (Fig. 7a). Deposit-like structures immunoreactive for DGC-components such as δ-sarcoglycan, dysferlin and integrin were identified (Fig. 7b, c and Additional file 2: Figure S3A) and focal sub-sarcolemmal enrichment of δ-sarcoglycan and integrin as well as chaperones (HSP70) were observed (Fig. 4a, Fig. 7b and Additional file 2: Figure S3A). Prompted by this finding, next transcript level of dysferlin, δ-sarcoglycan and integrin-beta-4 has been studied. Results showed nearly equal level for dysferlin whereas δ-sarcoglycan transcripts were ~ 50% increased and integrin-beta-4 transcripts were ~ 60% decreased (Additional file 2: Figure S3B). The combined findings might indicate that while some of the transcripts, such as δ-sarcoglycan, result in an elevated protein level for others, forced translation of stable or even decreased RNA leads to an increase in the corresponding proteins. Given that (i) the global proteomic signature of p.P104L CAV3 diseased quadriceps muscle indicated affection of DGC components such as dysferlin and (ii) UPR is also activated in diseased muscle as well as that (iii) further mass spectrometry studies did not identify the DGC components as elements of the proteins aggregates (Additional file 3: Table S1), effective targeting by UPR factors was hypothesized. Indeed, results of co-immunofluorescence studies revealed sarcoplasmic co-localization of GRP170 (a UPR-modulated chaperon) and dysferlin (Fig. 7c). However, although the architecture of the ER-Golgi network as organelles of protein glycosylation are altered and certain DGC components display vulnerability against p.P104L CAV3 protein expression, the myopathology does not affect proper glycosylation of α-dystroglycan as a key protein of the DGC (Fig. 7d).

## Discussion

Mutant CAV3 has been shown to mis-localize to the Golgi [8, 10] and cause ER-stress accompanied by activation of the UPR as a cellular defence mechanism, but the molecular consequences of the ER-Golgi disturbance as part of the CAV3 pathophysiology remained elusive. To systematically address this gap of knowledge and to further strengthen the understanding of pathophysiological processes in caveolinopathies, we comprehensively analysed quadriceps muscles from a well-characterized mouse model harbouring the dominant p.P104L mutation [11, 13].

### Vulnerability of the ER-Golgi system in p.P104L caveolinopathy

We performed morphological investigations of the ER-Golgi system, linked the structural perturbations to altered abundance of proteins involved in maintenance of organelle architecture and showed that prolonged perturbation of the ER-Golgi machinery—indicated by the altered protein abundance of ASNA1, EF1B, RCN2, UGGT1, TMEM43 and our immunohistochemical studies (Additional file 3: Table S1, Fig. 4 and Additional file 2: Figure S3)—results in the build-up of protein aggregates within diseased quadriceps muscles fibres. This observation is not only in line with the increased expression of protease inhibitors (i.e. ANT3, ILEUA, SPB6, MUG1 and A1AT4) but also with the increased abundance of overall ubiquitinated proteins (Fig. 6a). Concomitantly, increased expression of (co-)chaperones (HSP70/ HSPB1, HSPB2, HSPB7, BiP, SIL1; Additional file 3: Table S1 and Fig. 4) and of proteins involved in proteolysis such as CAPN/calpain (protease involved in the breakdown of misshaped proteins preventing toxic aggregation) and PSMA5 most likely represent a cellular defence mechanism antagonizing the build-up of toxic protein aggregates (Additional file 3: Table S1).

### Protein aggregation in p.P104L caveolinopathy

As elevated CAV3 level has been demonstrated to be beneficial for autophagic protein clearance [2], overall reduction of cellular CAV3 upon p.P104L CAV3 pathophysiology (Fig. 2) might influence the efficiency of cellular proteolysis via the autophagic system, an assumption supported by the build-up of protein aggregates. Our electron and CARS microscopy studies showed electron-dense myelin-like structures (most likely corresponding to protein aggregates) within the sarcoplasm and the sub-sarcolemmal region that could be inadequately processed substrates of the perturbed ER-Golgi machinery. Further molecular characterization of these aggregates revealed affection of a variety of proteins belonging to different subcellular compartments such as the sarcolemma, cytoskeleton and mitochondria (Additional file 3: Table S1) suggesting that the p.P104L mutation has as widespread effect on overall organelle integrity and function in muscle. Presumably, impaired protein processing leading to the accumulation of aggregates seems to be an upstream event in the pathological cascade of caveolinopathy. Therefore, rescuing or stabilizing the native conformations of proteins is an obvious therapeutic strategy. Over the last decade, small molecules known as chaperones have been shown to be effective in reducing levels of misshaped proteins, thus minimizing the accumulation of aggregates with toxic potential leading and reducing negative downstream pathological consequences [34] (Additional file 6: Figure S4).

### Vulnerability of further DGC components and the sarcolemma in p.P104L caveolinopathy

Constant production and Golgi mis-localization of mutant CAV3 most likely perturbs proper ER-Golgi function, which should also subsequently affect the substrate proteins processed within these compartments. Notably, DGC components are known to be processed within these compartments [35]. Indeed, our proteomic study revealed increased abundance of DGC components such as dysferlin, sarcoglycans, integrins, dystrophin and syntrophin (Additional file 3: Table S1, Fig. 7 and Additional file 2: Figure S3). Moreover, results of our immunological analyses indicated an abnormal localization of paradigmatic DGC components and showed, apart from immunoreactivity with the sarcolemma, a patchy distribution within the sarcoplasm and focal enrichment within the sub-sarcolemmal region (Fig. 7 and Additional file 2: Figure S3). However, DGC components have not been identified as constituents of the protein aggregates (Additional file 3: Table S1) and co-immunofluorescence studies showed co-localization of dysferlin with GRP170, a UPR-modulated chaperone suggesting that sarcoplasmic enrichment of DGC components is more likely based on prolonged processing/ chaperone mediated re-folding due to perturbed ER-Golgi function rather than on formation of aggregates.

Prompted by the altered architecture of the ER-Golgi network as cellular place of protein glycosylation as well as abnormal subcellular distribution of DGC components, glycosylation of α-dystroglycan as a paradigmatic DGC component has been studied but revealed no significant changes suggesting that p.P104L CAV3 pathophysiology targeting the ER-Golgi network does not have an impact on glycosylation of certain DGC key proteins. This assumption agrees with the identification of increased abundance of UGGT1, an ER-resident protein that selectively re-glucosylates unfolded glycoproteins, thus providing quality control for protein transport out of the ER (https://www.ncbi.nlm.nih.gov/gene/56886).

Hence, our combined data suggest that a vulnerability of the sarcolemma in caveolinopathy is not only based on the absence/ detrimental reduction of CAV3 [1]—moreover reflected by decrease of PTRF (Additional file 3: Table S1) but also on the aggregation of proteins important for sarcolemmal integrity and presumably strengthened by altered/prolonged processing of DGC components. In this context, direct functional relevance is indicated by aggregation of MAP3K2 playing a role in “caveolae kiss-and-run dynamics” [36] as well as by MYOC, a regulator of muscle hypertrophy through the components of dystrophin-associated protein complex [37]. However, our global proteomic profiling results also support the concept of sarcolemmal vulnerability, as a decrease of CAD13 and SLC41A3 was detected, proteins important for cell-cell contacts [38]. Increased abundance of DERM, regulating cell-cell adhesion by surface integrin binding seems to compensate affection of integrins (as part of the DGC) in the presence of mutated CAV3 (Additional file 2: Figure S3A). Also, the cellular decrease of CA3 supports this assumption as CA3 is a known serum biomarker for muscular dystrophy mirroring sarcolemmal fragility [39]. Compensatory mechanisms may counteract sarcolemmal vulnerability, which is supported by our proteomic results revealing the increased abundance of six plausible proteins (Additional file 3: Table S1). Among those proteins, PACN3 regulates internalization of sarcolemmal proteins. PACN3 overexpression impairs internalization of proteins such as GLUT1 and TRPV4 and increases the sarcolemmal level of those proteins. As GLUT1 facilitates the transport of glucose across the plasma membranes of mammalian cells [40] our data provide a molecular explanation for the recently described perturbed glucose metabolism in muscle cells upon p.P104L CAV3 expression [41]. TRPV4 contributes to cell volume control and plays a role in the modulation of muscle fibre atrophy [42]. ATP1A1 is increased (Additional file 3: Table S1) which controls the exchange of sodium and potassium ions across the sarcolemma, a process perturbed in Duchenne muscular dystrophy [43]. This accords with the activation of mechanisms antagonizing sarcolemmal vulnerability and improving cellular fitness.

### Vulnerability of cytoskeletal components in p.P104L caveolinopathy

Altered sarcolemmal-integrity as suggested (i) by our molecular characterization of the aggregates and (ii) by paradigmatic DGC components (linking the cytoskeleton to the basal lamina) showing partial mis-localization indicate downstream effects on cytoskeletal integrity. Indeed, our combined proteomic findings in p.P104L CAV3 diseased muscle are also indicative for cytoskeletal vulnerability (Fig. 4, Additional file 4: Figure S1 and Additional file 5: Figure S2 and Additional file 3: Table S1) linking the p.P104L CAV3 pathophysiology to impaired muscle fibre contraction: a variety of cytoskeletal proteins have been identified as constituents of the aggregates identified in muscle of diseased animals and results of our global proteomic studies showed a decrease of NEXN, MYH8, MYPT2 and MURC, whereas the decrease in TMOD4 and increases in 14-3-3-eta, COBL, HSPB7, GELS, MARCS, TUBA4A, TUBA8, DPYL2, SYNM, MAP4 and PDLIM3 (Additional file 3: Table S1) levels are suggestive of antagonizing mechanisms to avoid muscle fibre break-down.

### Vulnerability of mitochondria in p.P104L caveolinopathy

Cellular increase of CAV3 notoriously attenuates mitochondrial damage and cell death in (cardio) myocytes [44] thus indicating a functional relevance of CAV3 level for mitochondrial function. Notably, altered mitochondrial function in caveolinopathy influencing energy production and oxidative stress burden can be deduced from our global proteomic findings (Additional file 3: Table S1) and molecular characterization of aggregates identified in quadriceps muscles of p.P104L transgenic animals moreover revealed vulnerability of a total of 26 different mitochondrial proteins involved in diverse mitochondrial processes as well as in structural integrity (Fig. 6e and Additional file 3: Table S1). Vulnerability of mitochondrial proteins in p.P104L CAV3 pathophysiology accords with the identified ultra-structural abnormalities (Fig. 6c).

### Further pathophysiological insights into p.P104L caveolinopathy

The proteomic response to p.P104L mutant CAV3 expression moreover revealed an increased abundance of secreted proteins belonging to the extracellular matrix (ECM; in particular collagens) (Additional file 3: Table S1). Although these proteins are normally processed within the ER-Golgi machinery representing substrates, their increase might also arise from ECM accumulation/fibrosis, a well-known pathophysiological hallmark of intermediate to late stage muscular dystrophies [45]. However, mass spectrometry-based characterization of aggregates detected in p.P104L CAV3 diseased muscle identified several proteins regularly targeted for secretion as molecular constituents thus strengthening the hypothesis of impaired substrate processing and protein aggregate formation. Longer-term observation would be required to fully clarify whether the increase of ECM proteins exclusively reflects protein retention in the Golgi/ER associated with protein aggregate formation or incipient fibrosis or a combination thereof.

Additionally, results of our comparative proteome profiling revealed changes in annexins: annexins represent a class of dysferlin-interacting proteins [46] and modulate myoblast cell differentiation by promoting migration of satellite cells and, consequently, skeletal muscle differentiation [47]. Annexins are also involved in the modulation of inflammatory processes and membrane repair of human skeletal muscle cells [46, 48]. Thus, increased abundance of annexins may contribute to the regeneration of muscle fibres in caveolinopathies and may have therapeutic implications with respect to the development of annexin mimetics. Similarly, increased abundance of CD81, which plays an important role in the restitution of normal muscle architecture during muscle regeneration [49] might provide an attractive therapeutic target.

Apart from altered GLUT1 (see above) there is further indication for an altered glucose metabolism with changes in the abundance of PGM1, HK2 and RTN2 (Additional file 3: Table S1). Interestingly, regarding other pathways of potential importance, recent publications revealed that CAV3 directly interacts with the insulin receptor and that loss of CAV3 interferes with downstream insulin signalling and lipid uptake, implicating CAV3 as a regulator of the insulin receptor and regulator of lipid uptake [50]. Our proteomic findings support a role of CAV3 in lipid homeostasis by demonstrating decreased levels of FITM1, CES1D and EDF1 (Additional file 3: Table S1).

Finally, decreased abundance of SMYD1, EDF1 and PDLI5 might provide molecular insights into the nature of the dilatative cardiomyopathy described in this mouse model [13].

## Conclusions

Results of our combined morphological and biochemical studies confirm the previously reported ER-Golgi pathology caused by mislocalization of missense mutant CAV3 permanently stressing subcellular compartments. The organelle pathology is linked to impaired protein processing accompanied by the build-up of sarcoplasmic and sub-sarcolemmal protein aggregates. Thus, our study adds caveolinopathies to the growing list of secondary chaperonopathies [48]. Combined findings unravel substrates of the impaired ER-Golgi machinery (secondarily) disturbing sarcolemmal and mitochondrial integrity, the cytoskeleton and the ECM. On a more general note, our study represents a promising example of the suitability of applied proteomics to obtain deep insights into the underlying pathophysiology of diseases such as neuromuscular disorders [51].

## Additional files

Additional file 1: **Table S2.** List of antibodies used in this study. (DOC 53 kb)

Additional file 2: **Figure S3.** Further studies of substrates of the pathophysiology and quantification of immunoblot findings. (**A)** Immunohistochemistry of integrin α 5 and β 4 revealed a perturbed localization (similar to the result of δ-sarcoglycan staining depicted in Fig. 6B in the quadriceps muscle fibres of p.P104L mutant animals. **(B)** mRNA expression of *dysferlin* (*Dysf*), *integrin-β-4* (Igtb4) and *δ-sarcoglycan* (*Scgd*) normalized to *glycerinaldehyde-3-phosphate dehydrogenase* (*Gapdh*) in quadriceps muscle of 26 weeks old p.P104L CAV3 mutant animals and wild-type littermates (*n* = 4). N represents the number of independent samples measured in triplicates (*P < 0.05 vs. wild-type). Statistical significance between groups was analysed by an unpaired *t*-test using GraphPad software (San Diego, USA). Differences were considered significant with *P* < 0.05. All data are shown as means ±SEM. These studies revealed almost equal mRNA abundance for *Dysf* but decreased abundance for *Itgb4* and increased abundance for *Scgd* in p.P104L CAV3 mutants. **(C)** Quantification results of immunoblot findings (towards verification of proteomic findings). (PPTX 9155 kb)

Additional file 3: **Table S1.** Overview of all proteomic findings. (XLSX 915 kb)

Additional file 4: **Figure S1.** Cytoscape network analysis of the regulated proteins and in silico data from different perspectives: gene interrelations revealed by correlation weights, protein interactions as well as functional context given by the GO enrichment analysis. The nodes are linked based on their kappa score level (≥0.3), where only the label of the most significant term per group is shown. The node size represents the term enrichment significance. Functionally related groups partially overlap. (PPTX 559 kb)

Additional file 5: **Figure S2.** Further pathway analysis of altered proteins. PANTHER-based pathway analysis was performed for increased and decreased proteins separately and indicates vulnerability of cytoskeleton in p.P104L diseased quadriceps muscle fibres. (PPTX 237 kb)

Additional file 6: **Figure S4.** Illustration showing the proposed mechanisms of pathogenesis in Caveolinopathy associated with the p.P104L mutation. (PPTX 38 kb)

## Abbreviations

ACN: Acetonitrile
BCA: Bicinchoninic acid assay
CaCl_2_: Calcium chloride
CARS: Coherent anti-Stokes Raman scattering
CID: Collision induced dissociation
CK: Creatine kinase
Cys: Cysteine
DGC: Dystroglycan complex
DM: Distal myopathy
DTT: Dithiothreitol
ECM: Extracellular matrix
EDTA: Ethylenediaminetetraacetic acid
EM: Electron microscopy
ER: Endoplasmic reticulum
ERGIC: Endoplasmic reticulum Golgi intermediate compartment
FA: Formic acid
FDR: False discovery rate
Glu: Glutamic acid
GO: Gene ontology
GuHCl: Guanidinium chloride
H&E: Hematoxylin and eosin
HCK: HyperCKemia
HEK293: Human embryonic kidney cell line
HPLC: High-performance liquid chromatography
IAA: Iodoacetamide
IHC: Immunohistochemistry
LC-MS/MS: Liquid chromatography coupled to tandem mass spectrometry
LGMD: Limb Girdle Muscular Dystrophy
Met: Methionine
NaCl: Sodium chloride
PBS: Phosphate-buffered saline
PCR: Polymerase chain reaction
PMT: Photo multiplier tube
PVDF: Polyvinylidene difluoride
RMD: Rippling muscle disease
SDS: Sodium dodecyl sulfate
SR: Sarcoplasmic reticulum
TFA: Trifluoroacetic acid
TRIS: (Hydroxymethyl)aminomethane
UPR: Unfolded protein response
WT: Wild-type
