# Supplementary material for: Biochemical and pathological changes result from mutated Caveolin-3 in muscle
Source: Skelet Muscle. 2018 Aug 28;8:28. doi: 10.1186/s13395-018-0173-y (PMC6114045; doi:10.1186/s13395-018-0173-y)
Supplement: Supplementary file 1 — Table S2. List of antibodies used in this study. (DOC 53 kb) [file 13395_2018_173_MOESM1_ESM.doc]

| **Antibody** | **Company** | **Dilution for immunoblotting** | **Dilution for immunostaining** | **Concentration for immunoprecipitation** |
| --- | --- | --- | --- | --- |
| ATL1 | Merck Millipore | 1:500 | - | - |
| BiP | BD Transduction Lab | 1:1000 | 1:100 | - |
| CAPN2 | Abcam | 1:400 | 1:25 | - |
| CAV3 | Abcam | 1:400 | 1:50 | - |
| DYSF | Leica | 1:300 | 1:25 | - |
| DMD | Abcam | 1:200 | - | - |
| α-DG/ AF6868 | R&D Systems | 1:500 | - | - |
| β-DG/ AF6868 | R&D Systems | 1:500 | - | - |
| GM130 | Abcam | - | 1:50 | - |
| Golgin-97 | Genetex | - | 1:50 | - |
| GRP170 | Gentex | 1:1000 | 1:100 | - |
| HSP70 | Abcam | 1:1000 | 1:100 | - |
| ITGA5 | Cell signalling | - | 1:25 | - |
| ITGB4 | Cell Signalling | - | 1:25 | - |
| LC3 | Abcam | 1:1000 |  |  |
| p62 | Abcam |  | 1:100 | 4 µg |
| peIF2 | Cell Signalling | - | 1:100 | - |
| PPP2R1A | Invitrogen | 1:750 | - | - |
| RCN2 | Genetex | 1:500 | 1:50 | - |
| α-sarcoglycan | Leica | 1:500 | - | - |
| β-sarcoglycan | Leica | 1:500 | - | - |
| δ-sarcoglycan | Leica | 1:500 | 1:50 | - |
| SEC61B | Abcam | 1:300 | - | - |
| SERPINE | Novus Biologicals | - | 1:25 | - |
| SIL1 | Abcam | 1:500 | - | - |
| α-1-syntrophin | Abacm | 1:500 | - | - |
| UBB | Abcam | 1:750 | 1:100 | 4 µg |
