# Supplementary material for: Biochemical and pathological changes result from mutated Caveolin-3 in muscle
Source: Skelet Muscle. 2018 Aug 28;8:28. doi: 10.1186/s13395-018-0173-y (PMC6114045; doi:10.1186/s13395-018-0173-y)
Supplement: Supplementary file 2 — Figure S3. Further studies of substrates of the pathophysiology and quantification of immunoblot findings. (A) Immunohistochemistry of integrin α 5 and β 4 revealed a perturbed localization (similar to the result of δ-sarcoglycan staining depicted in Fig. 6B in the quadriceps muscle fibres of p.P104L mutant animals. (B) mRNA expression of dysferlin (Dysf), integrin-β-4 (Igtb4) and δ-sarcoglycan (Scgd) normalized to glycerinaldehyde-3-phosphate dehydrogenase (Gapdh) in quadriceps muscle of 26 weeks old p.P104L CAV3 mutant animals and wild-type littermates (n = 4). N represents the number of independent samples measured in triplicates (*P < 0.05 vs. wild-type). Statistical significance between groups was analysed by an unpaired t-test using GraphPad software (San Diego, USA). Differences were considered significant with P < 0.05. All data are shown as means ±SEM. These studies revealed almost equal mRNA abundance for Dysf but decreased abundance for Itgb4 and increased abundance for Scgd in p.P104L CAV3 mutants. (C) Quantification results of immunoblot findings (towards verification of proteomic findings). (PPTX 9155 kb) [file 13395_2018_173_MOESM2_ESM.pptx]

## Slide 1
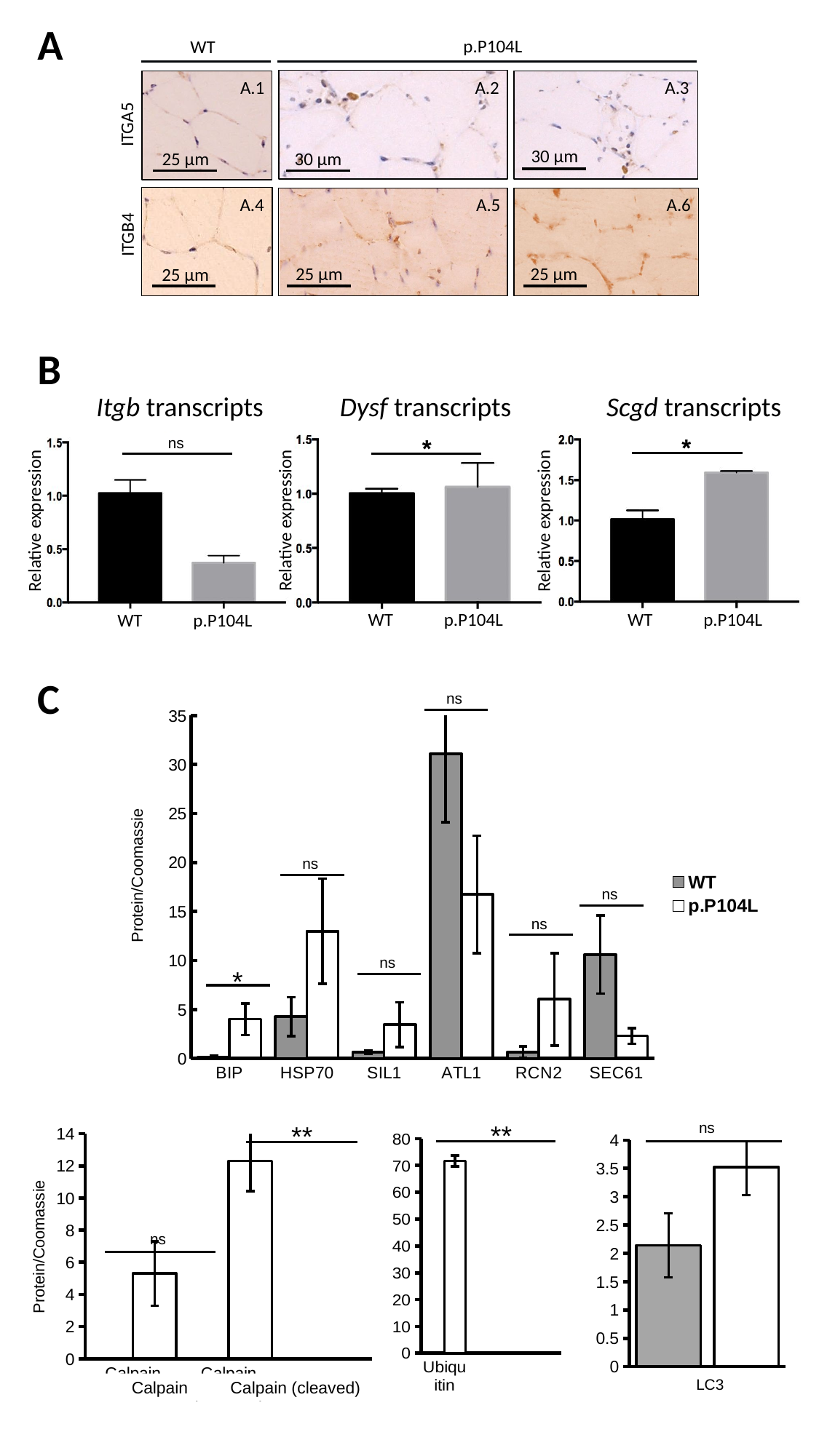

A
p.P104L
WT
A.2
A.1
A.3
ITGA5
30 µm
25 µm
30 µm
A.5
A.6
A.4
ITGB4
25 µm
25 µm
25 µm
B
Scgd transcripts
Dysf transcripts
Itgb transcripts
*
*
ns
Relative expression
Relative expression
Relative expression
WT
p.P104L
WT
p.P104L
WT
p.P104L
C
ns
### Chart
| Category | WT | p.P104L |
|---|---|---|
| BIP | 0.1708673149323697 | 4.022547410690573 |
| HSP70 | 4.269169510137275 | 12.99946278117983 |
| SIL1 | 0.6274610794374629 | 3.4533851581671975 |
| ATL1 | 31.09274721847871 | 16.7533292849816 |
| RCN2 | 0.6638074109899433 | 6.052811642867432 |
| SEC61 | 10.618457924062463 | 2.3147671155639373 |ns
Protein/Coomassie
ns
ns
ns
*
ns
**
**
### Chart
| Category | WT | p.P104L |
|---|---|---|
| Ubiquitin | 13.610680027576521 | 71.70036138327991 |
### Chart
| Category | WT | p.P104L |
|---|---|---|
| Calpain | 4.4 | 5.3 |
| Calpain (Sub-unit) | 4.113080577137841 | 12.306333697731825 |
### Chart
| Category | |
|---|---|
| WT | 2.143481309913253 |
| p.P104L | 3.526079833747822 |LC3
ns
Protein/Coomassie
 Calpain Calpain (cleaved)

## Slide 2
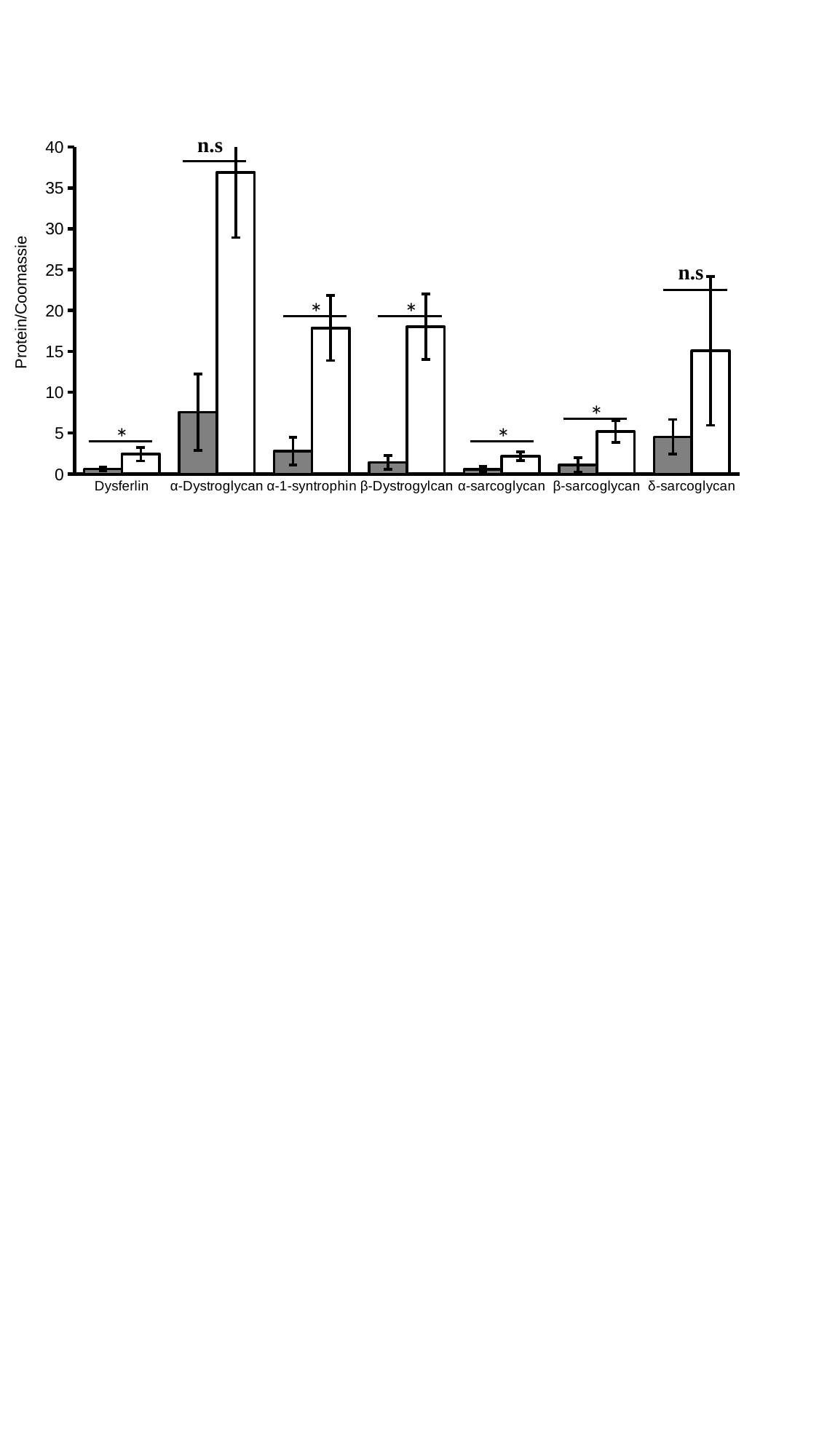

### Chart
| Category | WT | p.P104L |
|---|---|---|
| Dysferlin | 0.6080310576408191 | 2.421567120689638 |
| α-Dystroglycan | 7.545499741668283 | 36.90955629481051 |
| α-1-syntrophin | 2.773751806136057 | 17.860263878981943 |
| β-Dystrogylcan | 1.4132040717812788 | 18.012520310742577 |
| α-sarcoglycan | 0.5763467893095212 | 2.1561089217562306 |
| β-sarcoglycan | 1.1089421423944077 | 5.162338145660455 |
| δ-sarcoglycan | 4.534609614479064 | 15.062433311382415 |n.s
n.s
Protein/Coomassie
*
*
*
*
*
