# Supplementary figures and images for: Biochemical and pathological changes result from mutated Caveolin-3 in muscle
Source: Skelet Muscle. 2018 Aug 28;8:28. doi: 10.1186/s13395-018-0173-y (PMC6114045; doi:10.1186/s13395-018-0173-y)

## Slide 1
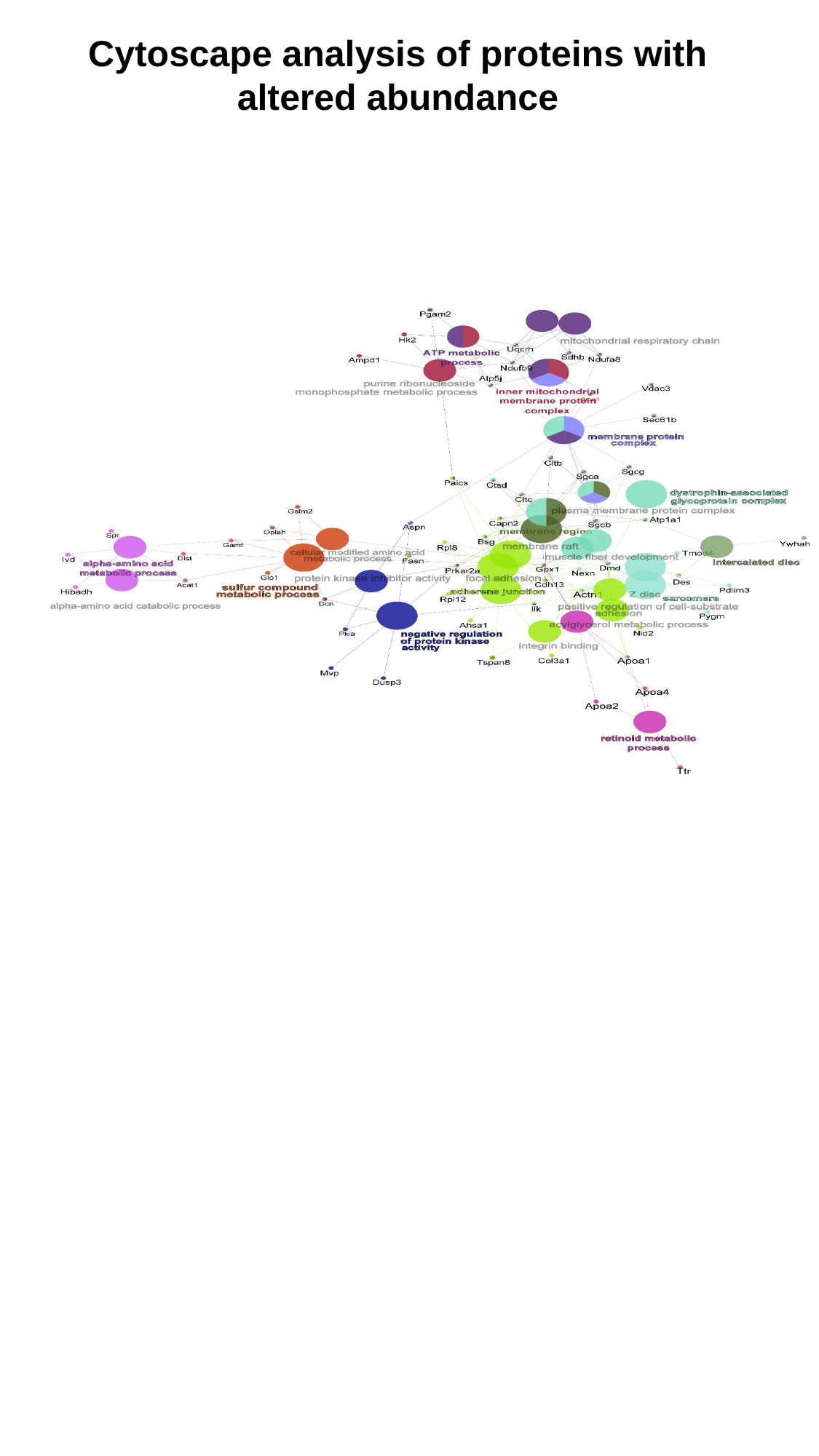

Cytoscape analysis of proteins with altered abundance

Supplement: Supplementary file 4 — Figure S1. Cytoscape network analysis of the regulated proteins and in silico data from different perspectives: gene interrelations revealed by correlation weights, protein interactions as well as functional context given by the GO enrichment analysis. The nodes are linked based on their kappa score level (≥0.3), where only the label of the most significant term per group is shown. The node size represents the term enrichment significance. Functionally related groups partially overlap. (PPTX 559 kb) [file 13395_2018_173_MOESM4_ESM.pptx]
