# Supplementary material for: Biochemical and pathological changes result from mutated Caveolin-3 in muscle
Source: Skelet Muscle. 2018 Aug 28;8:28. doi: 10.1186/s13395-018-0173-y (PMC6114045; doi:10.1186/s13395-018-0173-y)
Supplement: Supplementary file 5 — Figure S2. Further pathway analysis of altered proteins. PANTHER-based pathway analysis was performed for increased and decreased proteins separately and indicates vulnerability of cytoskeleton in p.P104L diseased quadriceps muscle fibres. (PPTX 237 kb) [file 13395_2018_173_MOESM5_ESM.pptx]

## Slide 1
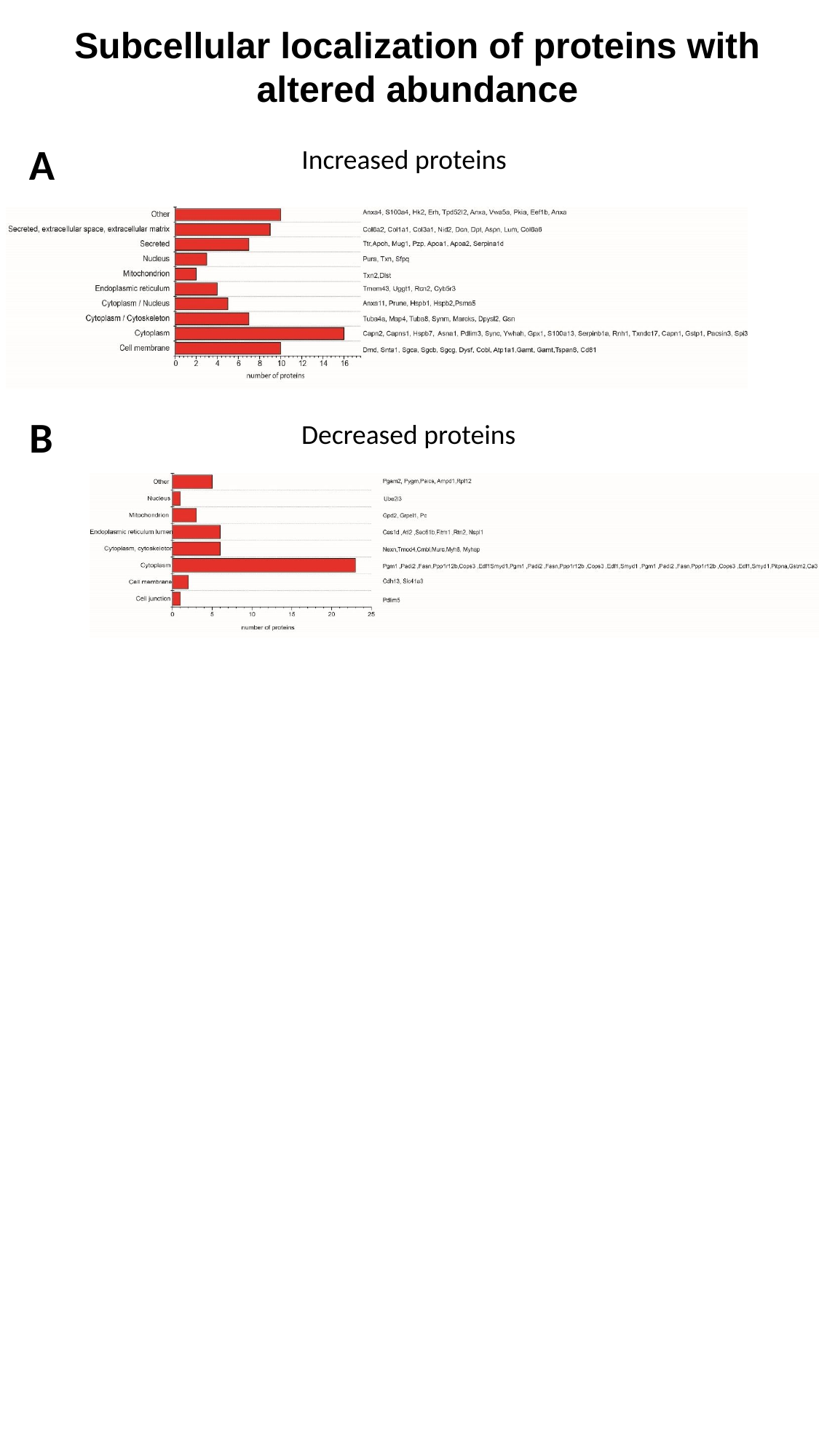

Subcellular localization of proteins with altered abundance
A
Increased proteins
B
Decreased proteins
