# Supplementary material for: Biochemical and pathological changes result from mutated Caveolin-3 in muscle
Source: Skelet Muscle. 2018 Aug 28;8:28. doi: 10.1186/s13395-018-0173-y (PMC6114045; doi:10.1186/s13395-018-0173-y)
Supplement: Supplementary file 6 — Figure S4. Illustration showing the proposed mechanisms of pathogenesis in Caveolinopathy associated with the p.P104L mutation. (PPTX 38 kb) [file 13395_2018_173_MOESM6_ESM.pptx]

## Slide 1
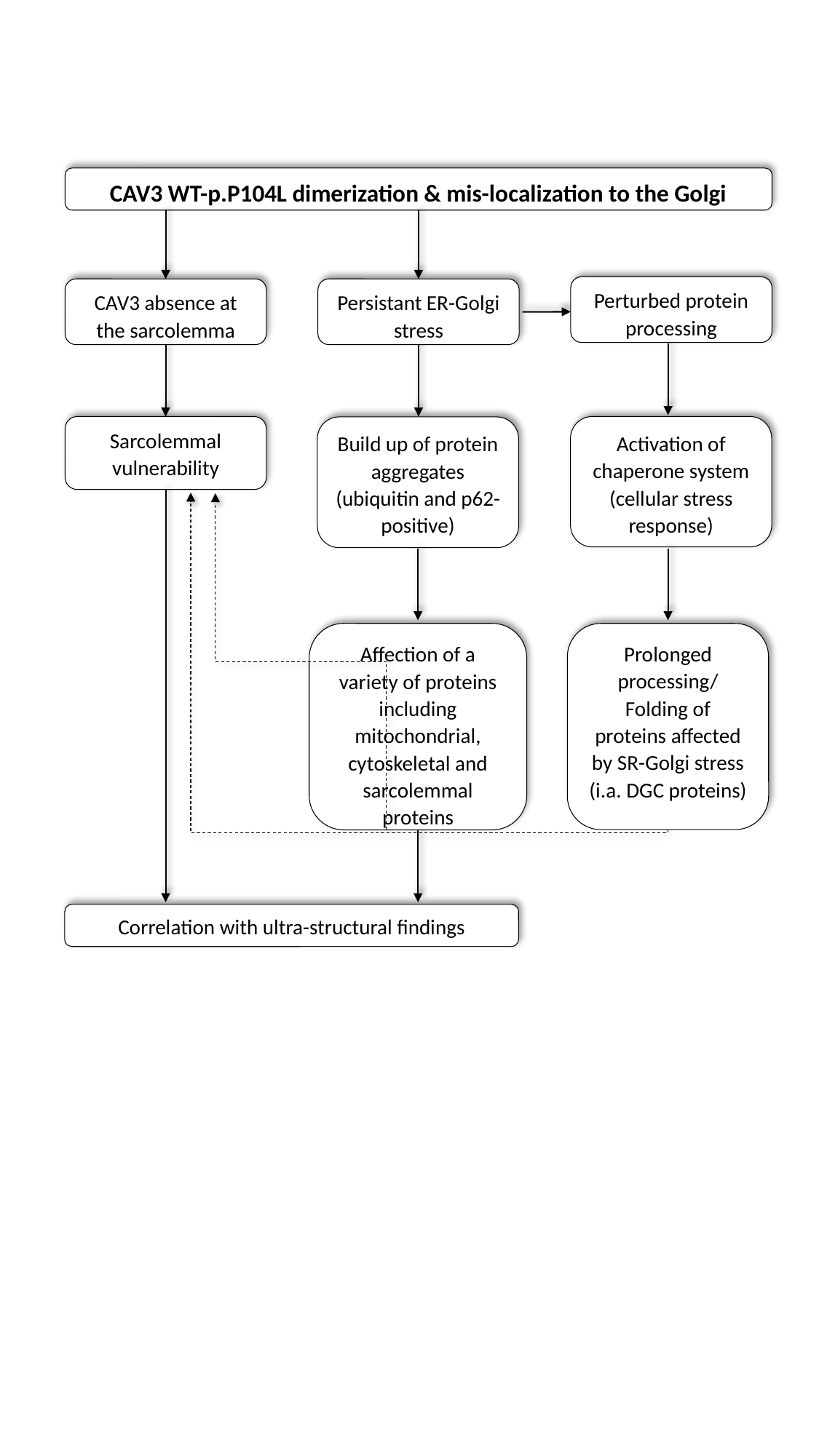

CAV3 WT-p.P104L dimerization & mis-localization to the Golgi
Perturbed protein processing
CAV3 absence at the sarcolemma
Persistant ER-Golgi stress
Sarcolemmal vulnerability
Activation of chaperone system (cellular stress response)
Build up of protein aggregates (ubiquitin and p62-positive)
Prolonged processing/ Folding of proteins affected by SR-Golgi stress (i.a. DGC proteins)
Affection of a variety of proteins including mitochondrial, cytoskeletal and sarcolemmal proteins
Correlation with ultra-structural findings
